# Lytic IFNγ is stored in cytotoxic granules and coreleased with granzyme B to mediate cytotoxic T lymphocyte killing

**DOI:** 10.1038/s41423-026-01391-1

**Published:** 2026-03-04

**Authors:** Xuemei Li, Claudia Schirra, Marie-Louise Wirkner, Szu-Min Tu, Chin-Hsin Lin, Meltem Hohmann, Yuan Gu, Llipsy Santiago, Julian Pardo, Iñaki Arretxe, Nadia Alawar, Abed Alrahman Chouaib, Ute Becherer, Po-Hsien Lee, Hao-Jen Hsu, Matthias W. Laschke, Cosima T. Baldari, Michael L. Dustin, Jens Rettig, Elmar Krause, Hsin-Fang Chang

**Affiliations:** 1 Cellular Neurophysiology, Center for Integrative Physiology and Molecular Medicine, (CIPMM), Saarland University, Homburg, Germany; 2 Department of Neurology, NHC Key Laboratory of Diagnosis and Treatment on Brain Functional Diseases, The First Affiliated Hospital of Chongqing Medical University, Chongqing, China; 3 Institute for Clinical and Experimental Surgery, Saarland University, PharmaScienceHub (PSH), Homburg, Germany; 4 Nanotoxicology and Immunotoxicology Unit (UNATI), IIS Aragón, Zaragoza, Spain; 5 Centro de Investigación Biomédica en Red en Enfermedades Infecciosas (CIBERINFEC), Instituto de Salud Carlos III (ISCIII), Madrid, Spain; 6 Centre for Biomedical Research of Aragon (CIBA), IIS Aragon/University of Zaragoza, Zaragoza, Spain; 7 Red RICOS de Terapias Avanzadas TERAV+ and CERTERA, Consorcio Estatal en Red para el desarrollo de Medicamentos de Terapias Avanzadas, IS Carlos III, Madrid, Spain; 8 Department of Biomedical Sciences and Engineering, Tzu Chi University, Hualien, Taiwan, China; 9 Department of Life Sciences, University of Siena, Siena, Italy; 10 Chinese Academy of Medical Sciences-Oxford Institute and Kennedy Institute of Rheumatology, Nuffield Department of Orthopaedics, Rheumatology and Musculoskeletal Sciences, University of Oxford, Oxford, UK

**Keywords:** Cytotoxic T lymphocyte, lytic IFNγ, granule secretion, immunological synapse, tumor immunity, Cytotoxic T cells, Interferons

## Abstract

Cytotoxic T lymphocytes (CTLs) eliminate target cells by forming immunological synapses and releasing effector molecules, including interferon gamma (IFNγ). However, how IFNγ contributes to cytotoxicity remains unclear. Here, we identify a subset of IFNγ stored within granzyme B⁺ cytotoxic granules (CGs) in activated mouse and human CTLs, which we term lytic IFNγ. This CG-associated IFNγ represents the primary pool released in a polarized manner at the immunological synapse together with canonical lytic molecules. Lytic IFNγ is present in tumor-infiltrating CTLs and is cosecreted with granzyme B (GzmB) in both soluble form and as part of supramolecular attack particles (SMAPs). Functional assays indicate that IFNγ contributes to CTL-mediated tumor cell death by acting in concert with granzyme B and perforin to increase cytotoxicity and promote apoptosis via the IFNγ–STAT1–caspase-3 pathway. CTLs lacking the vesicle priming factor Munc13-4 exhibit impaired release of both CGs and early-phase IFNγ. However, prolonged synapse engagement restores IFNγ secretion at distal membrane sites, revealing a second, nonpolarized IFNγ pool. Consistently, endogenous IFNγ is detected in both CG-enriched and multivesicular body (MVB)-enriched fractions. We propose that while lytic IFNγ is released from CGs at the synapse to directly promote target cell killing, nonpolarized IFNγ secretion originates from MVBs or small vesicles during sustained activation. Together, these findings reveal a previously unrecognized mechanism of IFNγ storage and release, establishing lytic IFNγ as a critical effector component of CTL cytotoxicity and antitumor immunity.

## Introduction

Cytotoxic T lymphocytes (CTLs) eliminate infected or malignant cells by releasing lytic molecules at immunological synapses [1, 2]. This process involves the polarization and secretion of cytotoxic granules (CGs) containing soluble perforin, granzyme B (GzmB), and supramolecular attack particles (SMAPs), which are particles encased by glycoproteins and extend cytotoxic engagement on target cells [3–5]. The secretion of these cytotoxic molecules is a tightly regulated process, with Munc13-4 being a critical priming factor for their release in CTLs and NK cells in both mice and humans [6, 7]. Loss-of-function mutations in Munc13-4 impair lytic granule exocytosis, leading to defective cytotoxic responses and life-threatening familial hemophagocytic lymphohistiocytosis [8].

Interferon γ (IFNγ) is a pivotal cytokine in adaptive immunity and is essential for immune regulation and antitumor defense [9–15]. It enhances the cytotoxic potential of CD8⁺ T cells, natural killer (NK) cells, and other effector cells [12, 16–18]. Both autocrine and paracrine IFNγ signaling are vital for T-cell cytotoxicity, and disruption of these pathways significantly impairs target cell killing [19]. While IFNγ also has context-dependent immunosuppressive effects, such as the modulation of T-cell exhaustion and the regulation of regulatory T-cell differentiation [20–22], emerging evidence indicates that IFNγ may directly contribute to cytotoxicity [23].

IFNγ is rapidly produced upon T-cell activation; however, its production is independent of mature synapse formation [24–26]. It is synthesized in the endoplasmic reticulum, processed in the Golgi apparatus, and subsequently released. In contrast to other cytokines, which are secreted multidirectionally, IFNγ has been shown to be polarized at the synapse [9, 27–29]. While cytotoxic synapses restrict killing to antigenic target cells, IFNγ exerts widespread effects, highlighting its multifaceted role in immunity [28, 30]. Prolonged synapse formation sustains IFNγ transcription and production [31]. Moreover, IFNγ has been identified within SMAPs from human CD8⁺ T cells, suggesting its potential role as an effector molecule [4]. However, the composition of murine SMAPs remains unexplored. Despite these findings, the dynamics of IFNγ storage and secretion in CD8⁺ T cells, including whether IFNγ is copackaged with other cytotoxic molecules, remain unclear.

In this study, we demonstrate that IFNγ is partially stored within GzmB⁺ cytotoxic granules in both human and murine CTLs, which we term “lytic IFNγ.” Lytic IFNγ is cosecreted with GzmB from the same granules at the immunological synapse in both soluble and SMAP-associated forms. Neutralizing secreted IFNγ in a T-cell–target cell coculture reduces the cytotoxic activity of mouse CTLs. Our findings reveal a previously unrecognized mechanism of IFNγ sorting as part of the cytotoxic machinery in CTLs and its additional contribution to antitumor immunity.

## Results

### IFNγ partially localizes to GzmB^+^ compartments in effector mouse CTLs

**Fig. 1:**
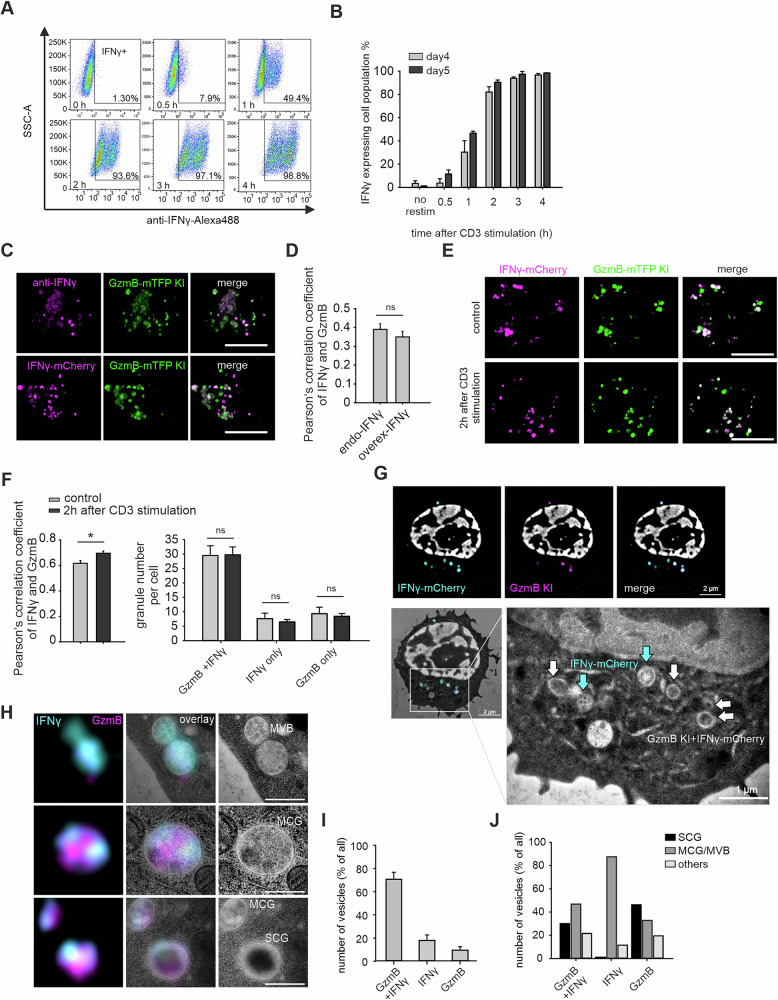
IFNγ partially localizes to GzmB compartments in mouse effector CTLs. **A** Day 5 effector mCTLs were stimulated on anti-CD3 antibody-coated plates, and intracellular IFNγ was stained and analyzed at the indicated time points via flow cytometry. **B** Kinetics of IFNγ expression in day 4 and day 5 effector CTL populations, derived from the analysis in (**A**). Three independent experiments were performed for FACS analysis. **C** Localization of IFNγ was analyzed in day 5 GzmB-KI effector CTLs via SIM. Endogenous IFNγ was detected with anti-IFNγ antibodies (upper panel), and IFNγ overexpression was visualized in GzmB KI cells expressing IFNγ-mCherry (lower panel). The cells were fixed on polyornithine-coated plates without restimulation. Scale bar, 5 µm. **D** Pearson’s colocalization analysis of endogenous IFNγ and overexpressed IFNγ with GzmB from **C** (*N* = 3 preparations; n_endo_ = 85 cells for endo, n_overexp_ = 47 cells). **E** SIM images of IFNγ-mCherry-overexpressing GzmB-KI effector CTLs plated on anti-CD3 antibody-coated plates for 2 hours. Scale bar, 5 µm. **F** Left: Pearson’s colocalization analysis of IFNγ-mCherry with GzmB before and after 2 hours of CD3 stimulation. **F** Right: the number of IFNγ⁺GzmB⁺ vesicles was also quantified under the conditions described in (**C**) (*N* = 3 preparations; n_control_ = 17 cells for endo, n_CD3_ = 18 cells). **G** CLEM analysis of IFNγ-mCherry-expressing day 5 GzmB-KI effector CTLs. The superresolution SIM images (top row) and the overlay with the corresponding electron microscopy (EM) image (bottom panel, left) are shown. In the magnified TEM image (bottom panel, right), the blue arrows point to IFNγ⁺ compartments, and the white arrows denote IFNγ⁺GzmB⁺ double-positive compartments. **H** Representative IFNγ⁺ and IFNγ⁺GzmB⁺ double-positive organelles cropped from three different mouse CTL images, as displayed in (**G**). SIM images (left), SIM/TEM overlay images (middle), and TEM images (right). Scale bar, 0.5 µm. **I** Quantification of IFNγ⁺GzmB⁺, IFNγ⁺, and GzmB⁺ organelles identified by CLEM as percentages. N = 5, n_cells_ = 27, n_organelles_ = 135. **J** Quantification of the different organelle fractions, defined by their expression profile and morphology (multicore granule, MCG; multivesicular body, MVB; single core granule, SCG, and others). *N* = 5, n_cells_ = 27, n_organelles_ = 135. The data are shown as the means ± SEMs. Statistical significance was determined via Student’s *t*-test: *p  <  0.05

To assess IFNγ distribution in CTLs, naive murine CD8⁺ T cells were activated with anti-CD3/CD28 Dynabeads and cultured with IL-2. On day 5, the cells were allowed to rest for 2 hours and were restimulated on anti-CD3-coated plates. Intracellular IFNγ expression increased rapidly, with approximately 50% of the cells expressing IFNγ⁺ within 1 hour, more than 90% of the cells expressing IFNγ within 2 hours, and nearly 100% of the cells expressing IFNγ within 4 hours (Fig. 1A and B).

Subcellular localization was examined in GzmB-mTFP knock-in CTLs [32] via structured illumination microscopy (SIM). Partial colocalization of endogenous and overexpressed IFNγ with GzmB⁺ compartments was observed (Fig. 1C) and quantified by Pearson’s correlation analysis (Fig. 1D). To investigate IFNγ sorting, we overexpressed IFNγ-mCherry in day 5 effector CTLs and analyzed its localization 8 hours after transfection, at which point IFNγ was largely removed from the Golgi [33] (Fig. 1E, upper panel). After 2 hours of stimulation, the colocalization of IFNγ and GzmB modestly increased (Fig. 1E, lower panel; Fig. 1F, left). However, the number of IFNγ⁺ and GzmB⁺ vesicles remained constant, suggesting that IFNγ is targeted to preexisting compartments (Fig. 1F, right).

Correlative light and electron microscopy (CLEM) revealed diverse morphologies of IFNγ⁺ compartments. The IFNγ⁺ and GzmB⁺ vesicles displayed typical CG features, including classical single-core granules (SCGs) and multicore granules (MCGs) [5] (Fig. 1G-H). Most IFNγ⁺ compartments (75%) were double positive for GzmB, whereas <20% were single positive for either IFNγ or GzmB (Fig. 1I). The IFNγ⁺ GzmB⁺ double-positive compartments predominantly presented CG structures, including SCGs and MCG/MVBs (categorized as one group), whereas the GzmB⁺ single-positive compartments were largely SCGs. In contrast, IFNγ⁺ compartments alone presented multivesicular body (MCG/MVB)-like structures distinct from those of SCGs. Conversely, structures that did not fit these classifications were categorized as “others” (Fig. 1J). Together, these findings indicate that IFNγ is produced upon CD3 stimulation and is partially sorted into GzmB⁺ CGs, including SCGs and MCGs.

### IFNγ and GzmB are coreleased at immunological synapses in mouse effector CTLs

**Fig. 2:**
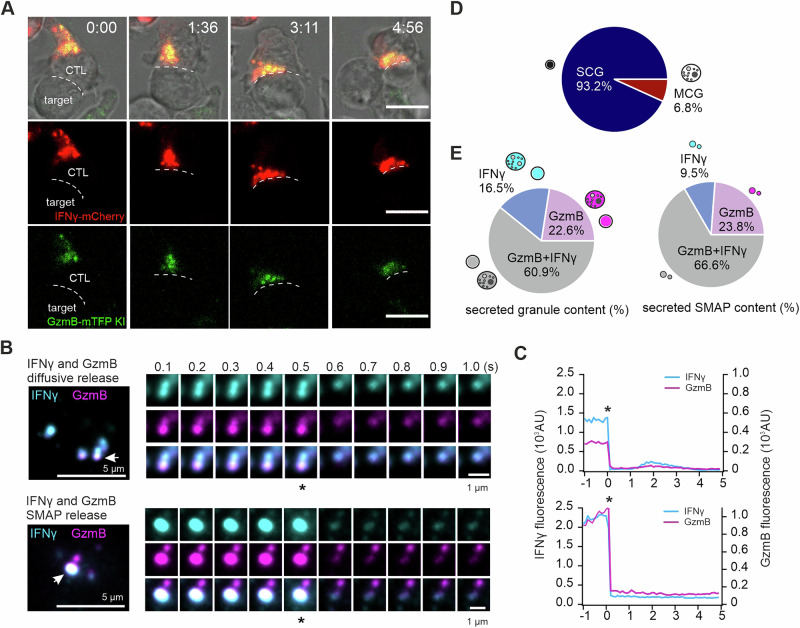
IFNγ and GzmB are coreleased at immunological synapses in mouse effector CTLs. **A** Live-cell confocal images of an IFNγ-mCherry-expressing GzmB-KI CTL forming a synapse with a P815 target cell. Scale bar, 5 µm. **B** On day 5, IFNγ-mCherry-expressing GzmB-KI CTLs were plated on anti-CD3-coated glass coverslips to induce granule secretion. The cells were perfused with 10 mM extracellular calcium buffer at RT, and secretion events were recorded via TIRFM. Snapshots show secreting granules (white arrows) at the synapse in individual cells (left panel); asterisks indicate the time frame when the fusion pore opens (right panels). Two secretion patterns are shown: IFNγ⁺GzmB⁺ double-positive granules with SMAPs (top) and diffusive release of IFNγ⁺GzmB⁺ granules (bottom). Images were acquired at 10 Hz. Scale bar, 1 µm. **C** Fluorescence intensity profiles of granule release from (B) over 5 s. A sudden decrease in IFNγ and GzmB fluorescence indicates fusion events. **D** Classification of granule secretion at the synapse. Granules exhibiting IFNγ⁺GzmB⁺ corelease, IFNγ-only release and GzmB-only release were categorized on the basis of SMAP deposition (MCG) or diffusive release (SCG) (N = 4 preparations; *n* = 274 cells). **E** Quantification of granules categorized by their secreted content of GzmB and/or IFNγ at the synapse (*N* = 4 preparations; *n* = 381 cells). Left, percentages of double-positive or single-positive granule secretion events. Right, proportion of secreted GzmB and IFNγ particles (*N* = 2 preparations; n_cell_ = 10, n_smaps_ = 21)

We next used confocal and total internal reflection fluorescence microscopy (TIRFM) to visualize real-time IFNγ release with high axial resolution at the T-cell synapse. IFNγ-mCherry was expressed in GzmB-mTFP KI effector CTLs to monitor IFNγ polarization upon target cell engagement. Live-cell confocal imaging revealed that IFNγ and GzmB polarized together toward the immunological synapse within 3 minutes of conjugation with P815 target cells (Fig. 2A; Movie S1).

To examine the specificity of IFNγ sorting to cytotoxic granules (CGs), we next tested whether its electrostatic properties contribute to granule targeting. IFNγ has a highly basic C-terminal region, suggesting that electrostatic interactions promote its incorporation into CGs. To address this, we generated IFNγ constructs with targeted C-terminal substitutions that selectively reduced the isoelectric point (pI) without altering overall protein folding, as assessed by AlphaFold3 structural prediction [34] (Fig. S1A). The basic sequence RKRKRSRC was replaced with either a neutral (IFNγ-A) or acidic (IFNγ-E) sequence, resulting in a marked reduction in the predicted pI.

Superresolution SIM revealed that, compared with wild-type IFNγ, both IFNγ-A and IFNγ-E mutants colocalized significantly less with serglycin and with GzmB⁺ cytotoxic granules (Figs. S1B-E). These results were confirmed under near-physiological expression conditions via the use of GzmB–tdTomato knock-in CTLs. In contrast, endogenous TNFα and IL-2 presented minimal colocalization with GzmB⁺ granules following target cell engagement (Fig. S1F), indicating that IFNγ is selectively enriched within CGs.

For TIRFM, CTLs were stimulated on anti-CD3-coated glass coverslips to form synapses, with additional extracellular calcium added to trigger granule release. Two distinct release patterns were observed: (1) IFNγ release in a soluble form or (2) IFNγ release accompanied by detectable particle deposition at the synapse. Soluble release has been shown to be characteristic of SCGs, whereas the deposition of particles is associated with MCGs [3–5] (Fig. 2B). Individual vesicle release events were visualized and analyzed. SCG release events resulted in full collapse of the vesicle at the synaptic membrane, marked by a sudden disappearance of diffusive IFNγ-mCherry and GzmB-mTFP fluorescence (Fig. 2B, upper panel). The fluorescence intensity profiles confirmed the rapid loss of signal at the time frame when the fusion pore opened (indicated by an asterisk; Fig. 2B and C, upper panel). In contrast, MCG released deposited IFNγ and GzmB after soluble release from the same vesicle, with persistent signals indicated by elevated baseline fluorescence postfusion (Fig. 2B, C, lower panels). The majority of the IFNγ signals in MCGs rapidly diffused away, highlighting the fact that both soluble IFNγ and SMAP-associated IFNγ are present in MCGs (Fig. 2C, lower panel). Quantitative analysis revealed that the majority of release events (93.2%) corresponded to SCGs, with only 6.8% representing MCGs (Fig. 2D). Among the released granules, 60.9% were positive for both IFNγ and GzmB, while 22.6% contained only GzmB, and 16.5% contained only IFNγ (Fig. 2E, left). The composition of SMAPs deposited at the synapse was similar to that of the granule contents: the majority encapsulated both IFNγ and GzmB (66.6%), whereas 23.8% contained only GzmB, and 9.5% contained only IFNγ (Fig. 2E, right). These findings highlight that the majority of IFNγ release at the synapse occurs in conjunction with GzmB, both in soluble and SMAP-associated forms. We term this population of IFNγ released alongside the cytotoxic mediator “lytic IFNγ”.

### IFNγ in cytotoxic granules exists in both soluble and SMAP-associated forms

**Fig. 3:**
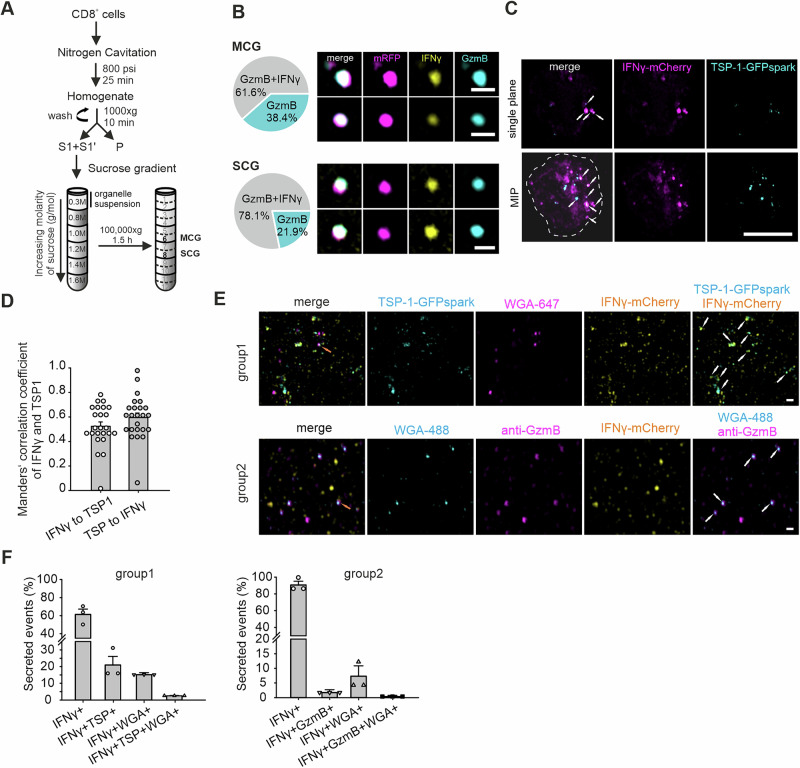
IFNγ in CGs is present in both soluble and SMAP-associated forms. **A** Schematic representation of the CG isolation procedure using discontinuous sucrose density gradient ultracentrifugation. Sucrose fractions enriched with multicore granules (MCGs; fraction 6) and single-core granules (SCGs; fraction 8) generated from Syb2-mRFT knock-in effector T cells were collected. **B** Left: Fraction of IFNγ⁺GzmB⁺ organelles within MCGs and SCGs (N_MCG_ = 3, n_MCG_ = 323, N_SC_ = 2, n_SCG_ = 32). Right: SIM images of representative MCGs and SCGs isolated from the respective fractions and stained with anti-IFNγ (yellow) and anti-GzmB-Alexa647 (cyan) antibodies. Syb2-mRFP (magenta) labels intact organelles. Scale bar, 0.5 µm. **C** SIM images of day 5 WT effector T cells coexpressing IFNγ-mCherry and TSP-GFPspark. Single-plane and maximum intensity projection (MIP) images are presented. The white dotted line outlines the cell footprint. The white arrows indicate regions of IFNγ and TSP-1 colocalization. Scale bar, 5 µm. **D** Manders’ colocalization analysis of IFNγ-mCherry and TSP-GFPspark signals from (**C**) (*N* = 3 preparations; *n* = 24 cells). **E** Day 5 effector T cells transfected with either IFNγ-mCherry and TSP-1-GFPspark (upper panel) or IFNγ-mCherry alone (lower panel) were plated on a supported bilayer coated with anti-CD3 antibodies to induce synapse formation. After 90 minutes of incubation at 37 °C, the secreted particles were analyzed for colocalization with SMAP markers, including granzyme B and WGA. The white arrows indicate double-positive particles, and the orange arrows indicate triple-positive particles. Scale bar, 0.5 µm. **F** Quantification of single-, double-, and triple-positive particles from (**E**) presented as percentages (*N* = 3 preparations; n_group1_ = 45 fields of view, with 18569 particles analyzed; n_group2_ = 41 fields of view, with more than 79994 particles analyzed). The data are presented as the means ± SEMs, with the distributions of individual data points shown

To investigate IFNγ packaging in CGs, we isolated granules from day 5 effector CTLs of Synaptobrevin2-mRFP knock-in (Syb2 KI) mice [35] via nitrogen cavitation and sucrose gradient centrifugation [36]. Syb2 is a marker of mature fusogenic CGs [35] in mouse CTL. This marker allowed us to discriminate CGs from other organelles after sucrose density gradient centrifugation [5]. We resolved two classes of CGs: MCGs in fraction 6 and SCGs in fraction 8 (Fig. 3A). The isolated granules retained mRFP membrane signals and GzmB content, indicating intact structures (Fig. 3B). Immunostaining for endogenous IFNγ revealed its presence in both fraction 6 and fraction 8 granules, which also contained GzmB. The quantification revealed that 61.6% of the MCGs and 78.1% of the SCGs were positive for IFNγ (Fig. 3B). Given the presence of IFNγ in MCGs, we investigated whether IFNγ was associated with insoluble SMAPs. Most day 5 effector cells (69%) retained IFNγ expression following dynabead activation without additional restimulation (Fig. S2, A and B). Coexpression of IFNγ-mCherry and TSP-1-GFPspark, a SMAP marker in WT effector CTLs, allowed us to examine IFNγ localization relative to the SMAP. High-resolution SIM revealed partial colocalization of IFNγ with TSP-1, including several small TSP-1 puncta encapsulated within IFNγ⁺ compartments (Fig. 3C, white arrows). Manders colocalization analysis revealed a greater colocalization of TSP-1 with IFNγ than did the reverse, which was consistent with the finding that TSP-1 was encapsulated within IFNγ⁺ compartments (Fig. 3D).

We next analyzed secreted IFNγ-SMAP associations. CTLs expressing IFNγ-mCherry with or without TSP-1-GFPspark were stimulated on supported lipid bilayers (SLBs) coated with ICAM-1 and anti-CD3 antibodies for 90 minutes, and the secreted material was stained with wheat germ agglutinin (WGA), a SMAP glycoprotein marker, and anti-GzmB (Fig. 3E). SIM imaging revealed that heterogeneously secreted materials, including IFNγ⁺ single-positive puncta, double-positive puncta, and triple-positive puncta, colocalized with TSP-1, WGA, or GzmB. Object-based analysis confirmed partial colocalization of all the markers, demonstrating that IFNγ is associated with SMAPs (Fig. 3F). Larger IFNγ-only puncta were likely to aggregate or be associated with alternative vesicle types, such as exosomes (Fig. 3E). Although IFNγ has been detected in SMAPs on the basis of proteomic data from human CTLs [4], its precise localization remains unclear. We isolated mouse SMAPs from the MCG fraction and observed that endogenous IFNγ puncta, detected via antibody staining, localized to the surface of GzmB⁺ SMAPs under nonpermeabilized conditions, whereas under permeabilized conditions, IFNγ colocalized with GzmB (Fig. S2C). These observations suggest that IFNγ is located both on the surface of SMAPs, associated with exosomes and encapsulated within SMAPs.

These results demonstrate that IFNγ exists in CGs both in soluble form and in SMAP-associated form, highlighting its diverse modes of packaging within effector CTLs.

### Lytic IFNγ localizes to cytotoxic granules and is cosecreted with GzmB in human effector CTLs

**Fig. 4:**
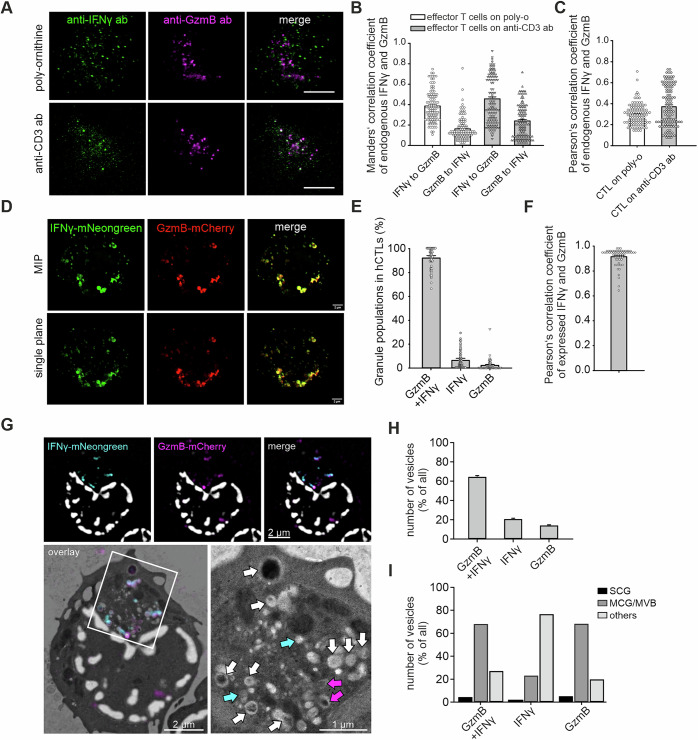
IFNγ partially localizes to GzmB compartments in human effector CTLs. **A** SIM images of day 5 human effector CTLs with endogenous IFNγ and GzmB stained with specific antibodies. After 10 minutes, the cells were fixed on poly-ornithine- or anti-CD3 antibody-coated glass coverslips. Scale bar, 2 µm. **B** Manders’ colocalization and **C** Pearson’s colocalization analysis of IFNγ-mNeonGreen and GzmB-mCherry signals from (**A**) (*N* = 3 donors; *n* = 105 cells). **D** SIM images of CTLs overexpressing IFNγ-mNeonGreen and GzmB-mCherry. The cells were fixed on polyornithine-coated coverslips without restimulation. Scale bar, 2 µm. **E** Pearson’s colocalization analysis of IFNγ-mNeonGreen and GzmB-mCherry signals from (**D**) (*N* = 3 donors; *n* = 51 cells). **F** Quantification of object-based colocalization of IFNγ-mNeonGreen and GzmB-mCherry granules (N = 3 donors; n_cells_ = 63). The data are presented as the means ± SEMs, with the distributions of individual data points shown. **G** CLEM analysis of IFNγ-mNeonGreen- and GzmB-mCherry-expressing day 5 human effector cells. SIM images (top row) and their overlay with the corresponding EM image (bottom panel, left) are shown. In the magnified TEM image (bottom panel, right), the blue arrows indicate IFNγ⁺ compartments, the magenta arrows indicate GzmB⁺ compartments, and the white arrows denote IFNγ⁺GzmB⁺ double-positive compartments. **H** Quantification of IFNγ⁺GzmB⁺, IFNγ⁺, and GzmB⁺ organelles identified by CLEM, expressed as percentages. **I** Quantification of the different organelle fractions, defined by their expression profile and morphology (multicore granule, MCG; multivesicular body, MVB; single core granule, SCG and others). *N* = 2, n_cells_ = 18, n_organelles_ = 268. The data are shown as the means ± SEMs. Statistical significance was determined via Student’s *t*-test: *p  <  0.05

To determine whether lytic IFNγ localization to CGs is conserved in human CTLs, we performed colocalization and secretion studies in day 5 effector CTLs generated from CD8⁺ peripheral blood mononuclear cells (PBMCs) stimulated with anti-CD3/CD28 beads. A subset (6.7%) of cells expressed IFNγ (Fig. S3A, B). SIM imaging of endogenous IFNγ and GzmB revealed partial colocalization in CTLs under unstimulated and stimulated conditions (Fig. 4A). Pearson’s and Manders’ analyses confirmed partial overlap, with higher Manders’ values for IFNγ with GzmB, suggesting both GzmB-associated and GzmB-independent IFNγ pools (Fig. 4B, C). While endogenous IFNγ and GzmB showed weak but detectable colocalization, the colocalization of hIFNγ-mNeonGreen and hGzmB-mCherry markedly increased, confirming the strong spatial association between these two molecules (Fig. 4D–F).

CLEM analysis revealed three vesicle populations under the same conditions as described above: double-positive (64.6%), IFNγ⁺ (20.9%), and GzmB⁺ (14.2%) (Fig. 4, G and H). Most of the double-positive granules and GzmB⁺ vesicles exhibited multigranular morphologies (MCG/MVB). IFNγ⁺ vesicles, which were mostly small and clear, were classified into a distinct “other” population (76.8%), whereas 23.2% had MCG/MVB-like structures (Fig. 4I).

**Fig. 5:**
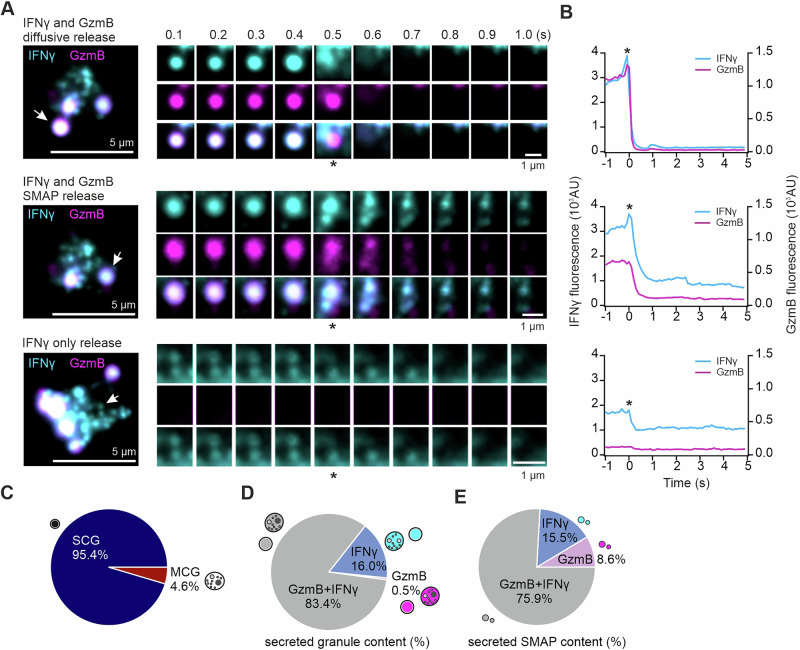
IFNγ and GzmB are coreleased at immunological synapses in human effector CTLs. **A** On day 5, CTLs transfected with IFNγ-mNeonGreen and GzmB-mCherry were plated on anti-CD3-coated glass coverslips to induce granule secretion for TIRFM analysis. Three secretion patterns were observed: IFNγ⁺GzmB⁺ double-positive granules with SMAPs (top), diffusive release of IFNγ⁺GzmB⁺ granules (middle), and IFNγ-only release (bottom). Snapshots show secreted granules (white arrows) at the synapses of individual cells (left panel); asterisks indicate the time frame at which the fusion pore opens. Images were acquired at 10 Hz. Scale bar, 1 µm. **B** Fluorescence intensity profiles of granule release from (**A**) over 5 s. A sudden decrease in IFNγ and GzmB fluorescence indicates fusion events. **C** Characterization of CG secretion at the synapse. Granules exhibiting IFNγ⁺GzmB⁺ corelease, IFNγ release only, and GzmB release only were categorized on the basis of SMAP deposition (MCG) or diffusive release (SCG) (*n* = 3 donors; *n* = 76 cells). **D** Quantification of GzmB and IFNγ secretion at synapses. Percentages of double-positive or single-positive granule secretion events (*N* = 3 donors; n_cells_ = 76; n_fusion events_ = 298). **E** Proportions of secreted GzmB and IFNγ particles (*N* = 3 donors; n_cells_ = 57; n_smaps_ = 98)

To assess secretion, human CTLs transfected with hIFNγ-mNeonGreen and hGzmB-mCherry were stimulated on anti-CD3-coated coverslips. Most secreted IFNγ colocalized with GzmB and was released diffusely with synchronous fluorescence loss (Fig. 5, A and B, upper panel; Movie S2). A subset of MCGs coreleased IFNγ and GzmB, depositing SMAPs that resulted in low fluorescence retention (Fig. 5, A and B, middle panel). A third IFNγ-only vesicle population also polarized and fused at the synapse, releasing IFNγ without GzmB (Fig. 5, A and B, lower panel). Quantitative analysis revealed that 95.4% of the fusion events were SCGs, with 4.6% MCGs (Fig. 5C). IFNγ fusion events were 83.4% GzmB-associated (lytic) and 16% nonlytic (Fig. 5D). Nonlytic IFNγ refers to the pool of IFNγ that is not colocalized with GzmB^+^ cytotoxic granules. Among the SMAPs, 75.9% contained both IFNγ and GzmB, whereas 15.5% and 8.6% contained only IFNγ and GzmB, respectively (Fig. 5E).

These findings demonstrate that human CTLs contain conserved lytic and nonlytic IFNγ populations, with IFNγ frequently copackaged and cosecreted with GzmB at the immunological synapse.

### Impaired lytic IFNγ release is accompanied by reduced cytotoxicity in mouse CTLs

**Fig. 6:**
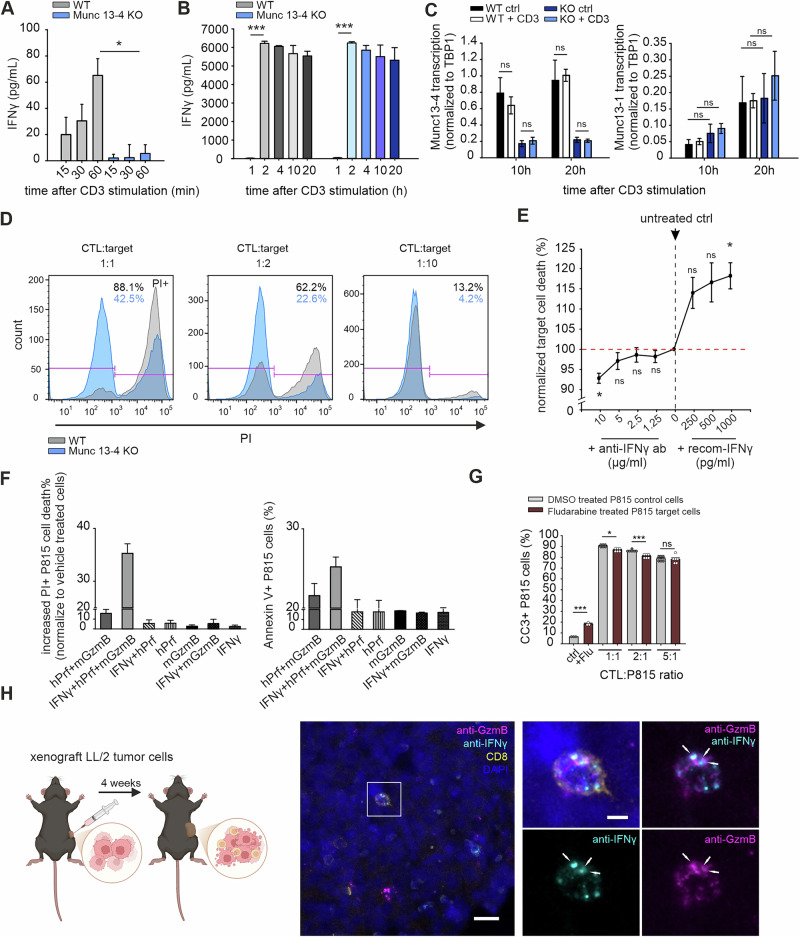
Impaired lytic IFNγ release leads to reduced cytotoxicity in mCTLs. **A**, **B** IFNγ release was measured in the supernatant via a LEGENDplex kit within 60 minutes (**A**) or an ELISA kit (**B**) within 1–20 hours. WT (gray) and Munc13-4 knockout (KO, blue) day 5 effector CTLs were plated on anti-CD3-coated plates. The supernatants were collected at the indicated time points (*n* = 3 for LEGENDplex analysis; *n* = 3 for ELISA analysis). **C** Normalized Munc13-1 and Munc13-4 mRNA transcription in WT and Munc13-4 KO CTLs at 10 h and 20 h after anti-CD3 stimulation (*n* = 3). **D** FACS-based killing assay of WT (gray) and Munc13-4 KO (blue) T cells. CTLs were cocultured with P815 target cells at the indicated T-cell-to-target ratios for 3 h. Dead cells were stained with propidium iodide (PI) to evaluate cell death. One representative experiment from 4 independent preparations is shown. **E** Effect of anti-IFNγ antibody neutralization (left quadrants) or the addition of recombinant IFNγ protein (right quadrants) on CTL-mediated killing. Day 4 effector WT CTLs were mixed with P815 target cells as described in (**D**). Dead cells were stained with PI. Untreated control cells are shown at the midpoint of the curve (*N* = 3). **F** FACS-based killing assay of recombinant IFNγ with other lytic molecules on P815 target cells. P815 cells were preincubated with recombinant IFNγ (10 ng/ml) overnight and subsequently treated with sublytic human perforin (hPerf) and mouse granzyme B (mGzmB; 600 nM) for 4 h. Cell death was quantified by flow cytometry and is expressed as the increase in cell death (%) relative to the basal level in vehicle-treated controls. The data represent the means ± SDs from two independent experiments; a total of four independent experiments were performed with comparable trends. **G** FACS analysis of the killing assay in target cells with IFN–STAT1 blockade. P815 cells were pretreated with 1 µg/ml fludarabine for 12 h. Day 3 activated CTLs were cocultured with these target cells for a 4 h killing assay. Dead cells were labeled with cleaved caspase-3 dye. Data from six replicates across two independent preparations are shown. **H** Confocal images of tumor-infiltrating CD8⁺ T cells in a xenograft tumor model. LLC/2 lung cancer cells were implanted subcutaneously into a mouse (left). After four weeks, the tumors were harvested, fixed, and stained with anti-CD8-PE, anti-IFNγ-Alexa488, and anti-GzmB-Alexa647 antibodies to label infiltrating CTLs. The arrows indicate colocalization of IFNγ and GzmB within a CD8⁺ T-cell in the inset (right). Scale bars: 20 µm (overview) and 5 µm (inset). The data are presented as the means ± SEMs. Statistical significance was determined via Student’s *t* test: *p  <  0.05, *p  <  0.001; ns indicates nonsignificance

To assess the contribution of lytic IFNγ to CTL cytotoxicity, we first investigated the molecular mechanisms underlying IFNγ release, focusing on Munc13-4, a priming factor essential for CG exocytosis in CTLs [37]. CTLs from Munc13-4 KO mice (Jinx mice [38]), in which the mutated Munc13-4 gene produces a nonfunctional protein, were compared with WT cells. The IFNγ mRNA expression levels of day 5 effector WT and KO cells were similar (Fig. S4A), followed by a comparable upregulation of the IFNγ gene expression profile upon anti-CD3 stimulation. IFNγ reached maximal expression after 2 hours of stimulation (Fig. S4B), with no significant differences observed between WT and KO cells. To assess IFNγ secretion, we stimulated WT and Munc13-4 KO effector CTLs on anti-CD3-coated plates and performed a time-course analysis over a 20-hour period. IFNγ secretion was significantly impaired in KO cells during the first hour of stimulation. A flow cytometry–based LEGENDplex cytokine assay, which uses bead-based immunoassay technology to simultaneously quantify multiple cytokines in cell culture supernatants, was performed to measure IFNγ secretion following CD3 stimulation. During the first 60 minutes of stimulation, WT CTLs produced measurable levels of IFNγ (up to 70 pg/mL), whereas KO CTLs exhibited severe impairment, releasing only minimal IFNγ (up to 10 pg/mL) into the supernatant (Fig. 6A). However, after 2 hours of stimulation, ELISA revealed that IFNγ concentrations in both WT and KO cultures reached comparable levels (approximately 62,000 pg/mL), indicating that sustained stimulation restored IFNγ secretion in KO CTLs (Fig. 6B). Constitutive IFNγ release in KO cells exhibited a slight but not significant increase in secretion in the absence of stimulation (Fig. S4C).

Notably, overexpression of IFNγ-mCherry in effector CTLs resulted in high levels of constitutive secretion in the absence of anti-CD3 antibody stimulation in WT cells (Fig. S5). This finding suggests that IFNγ release can occur independently of stimulation when protein expression levels are elevated, implying the presence of an additional IFNγ vesicle population distinct from the lytic IFNγ granule population. Since Munc13-4 KO cells exhibit normal IFNγ mRNA expression, the restoration of IFNγ release after 2 hours of stimulation indicates the involvement of a compensatory, Munc13-4-independent mechanism during prolonged activation. We assessed Munc13-1 expression in WT and Munc13-4 KO CTLs at 10 and 20 hours post-stimulation to examine its potential to compensate for Munc13-4 loss, given the shared priming function of the cytotoxic granules [37]. qPCR analysis revealed no significant differences in Munc13-1 expression between genotypes (Fig. 6C), suggesting that late-phase IFNγ secretion arises from nonsynaptic sources rather than from compensatory upregulation of Munc13-1. Furthermore, our TIRFM imaging revealed that CG secretion occurs predominantly during early stimulation, within 10 minutes of synapse formation. Granule secretion was rarely detected, despite the majority of CGs remaining polarized at the synapse (Figs. 4 and 5). This early-phase IFNγ release appears to be crucial for target cell cytotoxicity. Although Gzm and perforin are known to be the major contributors to granule-mediated killing, the fact that IFNγ is coreleased with GzmB and is concentrated at the synaptic cleft suggests that IFNγ may also contribute directly or indirectly to cytotoxicity. Consequently, Munc13-4 KO CTLs, which are deficient in CG and early IFNγ secretion, exhibited reduced killing of P815 target cells in 2-hour coculture assays (Fig. 6D). Moreover, neutralizing antibodies against IFNγ further decreased the killing efficiency of WT CTLs, whereas recombinant IFNγ increased the cytotoxicity of WT cells, demonstrating that soluble IFNγ contributes directly as an effector molecule to acute target cell killing (Fig. 6E). Notably, the recombinant IFNγ protein alone did not induce cell death, suggesting that IFNγ requires other lytic molecules to synergize with its cytotoxic effects (Fig. S4D). To investigate synergy, we performed killing assays in which IFNγ, GzmB, and perforin were combined. While IFNγ alone had no detectable effect, the combination of IFNγ, GzmB, and perforin resulted in a consistent trend toward enhanced target cell killing compared with any two- or single-molecule combination across four independent experiments, although the differences did not reach statistical significance. Propidium iodide (PI)and Annexin V staining confirmed this synergistic effect (Fig. 6F). To test whether IFNγ enhances cytotoxicity via caspase-dependent pathways, we blocked STAT1 signaling in P815 cells via fludarabine, which reduces STAT1 protein and mRNA levels [39, 40]. STAT1-inhibited target cells exhibited significantly reduced cleaved caspase-3 (CC3)-positive death in 4-hour killing assays, indicating that IFNγ induces apoptosis through the IFN–STAT1 pathway in addition to granule-mediated killing (Fig. 6G). Finally, to assess the physiological relevance of lytic IFNγ in CTLs, we implanted LL/2 tumor cells into the flanks of syngeneic mice and imaged tumor-infiltrating CTLs four weeks after implantation. Tumor slices were stained with antibodies against CD8, GzmB, and IFNγ. Consistent with our in vitro findings, a subset of GzmB⁺ cytotoxic granules contained IFNγ. These results support a functional role for lytic IFNγ in effector CTLs within the tumor microenvironment (Fig. 6H). Together, these findings show that the early, Munc13-4–dependent release of lytic IFNγ is critical for CTL cytotoxicity. Killing assays demonstrated that IFNγ contributes to target cell death both through direct granule-mediated cytotoxicity, which synergizes with GzmB and perforin, and, in part, via the IFN–STAT1–caspase apoptotic pathway, which may be particularly important for mediating tumor cell death.

### Prolonged synaptic engagement promotes distal IFNγ secretion in mouse and human CTLs

**Fig. 7:**
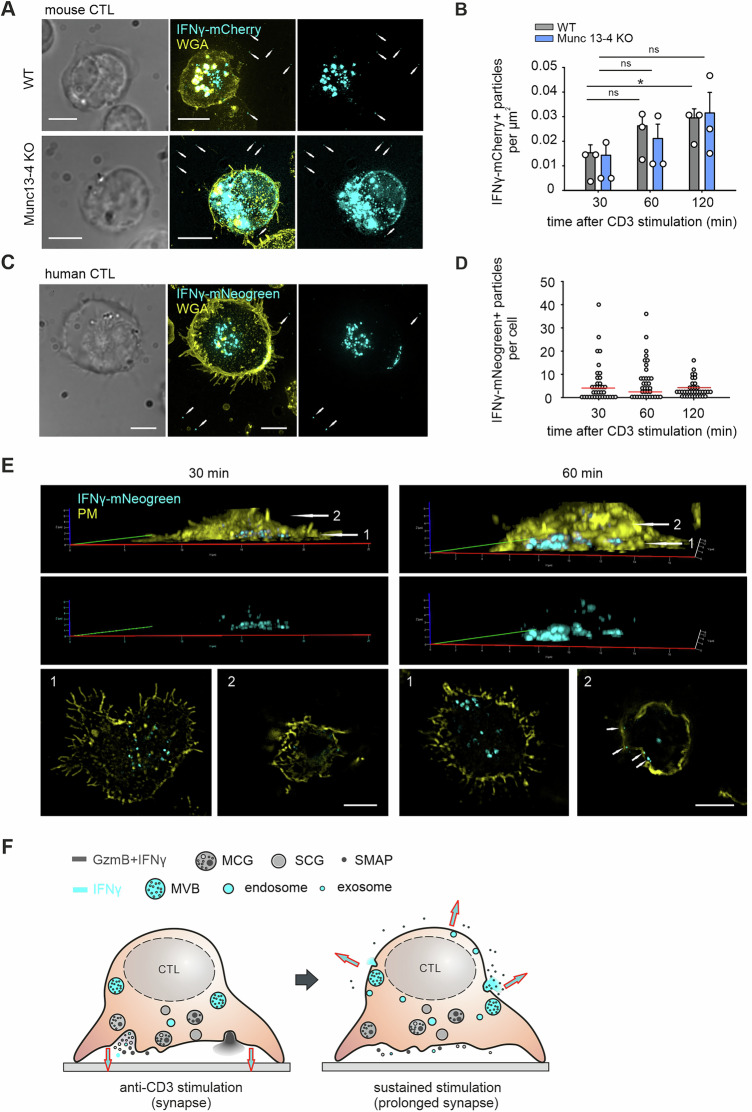
IFNγ secretion at the distal plasma membrane in mouse and human CTLs. **A** Representative SIM images of day 5 effector WT and Munc13-4 KO mouse CTLs fixed after 60 minutes on anti-CD3 antibody-coated coverslips. CTLs were transfected with IFNγ-mCherry (cyan) and stained with WGA (yellow) to mark the plasma membrane. The white arrows indicate secreted IFNγ-mCherry⁺ particles outside the cells. Scale bar, 5 μm. **B** Quantification of secreted IFNγ-mCherry⁺ particles in the extracellular regions from (**A**) (*N* = 3 mice; *n* = 25–30 areas per group). **C** Representative SIM images of day 5 effector human CTLs fixed after 60 minutes on anti-CD3 antibody-coated coverslips. CTLs were transfected with IFNγ-mNeonGreen (cyan) and stained with WGA (yellow) to mark the plasma membrane. The white arrows indicate secreted IFNγ-mNeonGreen⁺ particles outside the cells. Scale bar, 5 μm. **D** Quantification of secreted IFNγ-mNeonGreen⁺ particles from (**C**) at the indicated time points (*n* = 3 donors; *n* = 36 cells per time point). **E** Day 5 effector human CTLs transfected with IFNγ-mNeonGreen were fixed after 30 minutes (left) and 60 minutes (right) on anti-CD3 antibody-coated coverslips. The plasma membranes were stained with WGA. The upper panels show 3D reconstructions. Two distinct z-positions, namely, synaptic (1) and distal (2), are defined by arrows. The lower panels show the corresponding single-plane xy images. The white arrows in the distal layer (position 2) at 60 minutes indicate IFNγ⁺ vesicles attached to the plasma membrane away from the synapse. Scale bar, 5 μm. **F** Schematic illustration of IFNγ secretion in CTLs. Lytic IFNγ is packaged within CGs containing GzmB, including SCGs and MCGs, and is also associated with SMAPs. IFNγ is predominantly coreleased with GzmB at immunological synapses (left). Prolonged synapse formation results in an additional population of IFNγ being released at distal plasma membrane sites, where IFNγ is secreted from MVBs or via small vesicles. The red-bordering arrows indicate the direction of IFNγ secretion

To investigate the spatial dynamics of lytic and nonlytic IFNγ release, we analyzed day 5 effector mouse and human CTLs transfected with IFNγ-mCherry or IFNγ-mNeongreen. Upon anti-CD3 stimulation on glass coverslips, CTLs form tight synapses that retain secreted material within the synaptic cleft. In both WT and Munc13-4 KO mouse CTLs, IFNγ⁺ particles were also detected outside the synaptic area, suggesting secretion from the distal plasma membrane (Fig. 7A). While most soluble IFNγ diffused into the supernatant, a minority of IFNγ⁺ particles, possibly exosome-associated, remained detectable on the surface. Quantification confirmed comparable particle release between WT and KO cells over 2 hours, supporting the ELISA results that distal secretion persists in Munc13-4-deficient CTLs (Fig. 7B). Similar distal IFNγ particle release was observed in human CTLs, with 2–4 particles detected per cell within a 30 µm radius after 2 hours of stimulation (Fig. 7, C and D).

We next asked whether IFNγ⁺ vesicles actively polarize to distal regions. 3D-SIM imaging at 30 and 60 min post-stimulation revealed IFNγ accumulation at the synapse early (30 min), followed by a shift of IFNγ⁺ vesicles to the distal membrane at 60 min (Fig. 7E; Fig. S6A, B). To determine whether nonpolarized IFNγ originates from preexisting vesicle pools or newly synthesized protein, we treated IFNγ–mCherry–expressing mouse CTLs with brefeldin A to block intracellular protein transport 6 h after transfection, a time point at which IFNγ–mCherry expression is established and partially sorted from the Golgi. Consistent with previous results, DMSO-treated control cells displayed rapid polarization of IFNγ⁺ vesicles toward synapses within 30 min, followed by modest accumulation of small IFNγ⁺ vesicles at the distal membrane after 60 min (white arrows). This pool of IFNγ⁺ vesicles is likely fusogenic to the plasma membrane. In contrast, brefeldin A–treated cells presented slightly enlarged cytoplasmic IFNγ⁺ aggregates (orange arrows), indicating that newly synthesized IFNγ was retained in the Golgi region due to trafficking inhibition. These IFNγ⁺ aggregates were nonfusogenic, even when positioned close to the plasma membrane, and were distinguishable from the individual, fusogenic IFNγ⁺ vesicles observed in control cells (Fig. S6C). These observations suggest that most nonpolarized IFNγ⁺ vesicles arise from newly synthesized IFNγ rather than preexisting vesicle pools. To examine the source of IFNγ release in CTLs, we isolated MVBs and cytotoxic granules via cellular organelle fractionation, as described in Fig. 3A (see Materials and Methods). The MVB-enriched fraction was identified in fraction 4, whereas the classic single-core cytotoxic granule (SCG)-enriched fraction was found in fraction 8. Endogenous IFNγ was detected by antibody staining in GzmB-tdTomato KI–derived mouse CTLs. MVBs were positive for the exosome marker CD81, whereas SCGs contained GzmB, which was absent in MVBs. Both vesicle types contain IFNγ, supporting their roles as sources of IFNγ secretion in CTLs—potentially with SCGs releasing IFNγ at the immunological synapse and MVBs contributing to secretion at distal membrane sites (Fig. S6D). This polarization, observed in both mouse and human CTLs, was absent at earlier time points and likely reflects a distinct, nonlytic IFNγ pool mobilized during prolonged stimulation. These results reveal that, in addition to synaptic secretion, IFNγ is also released from the distal membrane in both mouse and human CTLs, particularly under sustained activation.

We propose that IFNγ sorted into cytotoxic granules constitutes the primary pool released at the immunological synapse together with canonical lytic molecules, thereby contributing to target cell killing in a polarized manner. Upon prolonged synaptic stimulation, a second pool of IFNγ is released distal to the immunological synapse, either within small vesicles or via exosomes derived from multivesicular bodies (MVBs), in a Munc13–4–independent manner. Together, these findings support a dual-pathway model of IFNγ secretion in CTLs, comprising an early, synaptic granule-associated pathway and a delayed, distal secretion pathway engaged during sustained activation (Fig. 7F).

## Discussion

Effector CTLs are central to antitumor immunity, exerting their function through both cytotoxic granule (CG)-dependent killing and cytokine secretion. Among these cytokines, IFNγ plays a well-established role in shaping the tumor microenvironment by enhancing antigen presentation and modulating immune responses [17, 19, 20, 30, 41]. However, whether and how IFNγ directly contributes to target cell cytotoxicity has remained unresolved.

Here, we define “lytic IFNγ” as a distinct pool of IFNγ that is sorted into cytotoxic granules and coreleased with canonical lytic molecules, such as granzyme B, at the immunological synapse. Functionally, we demonstrate that recombinant IFNγ alone exhibits minimal cytotoxic activity, whereas IFNγ in combination with granzyme B and perforin results in greater target cell killing than does granzyme B/perforin alone. This synergistic effect is abolished in Munc13-4–deficient CTLs, which are unable to release cytotoxic granules yet retain distal IFNγ secretion and nevertheless display profoundly impaired killing capacity. Furthermore, interference with IFNγ–STAT1 signaling in target cells reduces CTL-mediated target cell killing, indicating that IFNγ contributes to cytotoxicity through IFNγ receptor–dependent activation of apoptotic signaling pathways. Together, these findings support a model in which synaptically released IFNγ acts in concert with classical lytic effectors to potentiate CTL-mediated target cell death, likely through IFNγ–STAT1–dependent apoptotic pathways.

The precise trafficking mechanisms of IFNγ within T cells remain incompletely understood. One potential mechanism is linked to the isoelectric point (pI), as basic proteins such as IFNγ may interact electrostatically with serglycin. In complex with GzmB, which carries mannose-6-phosphate (M6P), IFNγ may be directed toward granule formation at the trans-Golgi network via the mannose-6-phosphate receptor (MPR) [42, 43]. In contrast, neutral or acidic cytokines such as IL-2 and TNFα may preferentially sort outside CGs, explaining the selective incorporation of IFNγ into cytotoxic granules. Consistent with this model, experimentally reducing the pI of IFNγ through targeted C-terminal substitutions significantly impaired its localization to cytotoxic granules in both IFNγ-A and IFNγ-E mutants, providing direct support for a pI-dependent sorting mechanism.

Our data reveal that IFNγ is prestored in both CGs and MVBs, giving rise to functionally distinct “lytic” and “nonlytic” pools. Using Munc13-4–deficient CTLs, we show that early-phase IFNγ release depends on CG exocytosis in a Munc13-4-dependent manner, whereas prolonged stimulation triggers secretion from distal membrane sites in a Munc13-4-independent manner. Brefeldin A treatment abolished distal release, indicating that newly synthesized IFNγ is sorted into MVBs and secreted via nonpolarized pathways. This secondary, nonlytic pool may serve as an alternative route when prestored granules are saturated, supporting continuous immune signaling during extended synaptic engagement [44–46].

Lytic molecules such as GzmB and perforin are secreted in a highly polarized manner to ensure specificity at the immunological synapse. Similarly, coreleased synaptic “lytic IFNγ” is concentrated at the cleft, suggesting a potentially distinct functional role from that of IFNγ released at the distal membrane. IFNγ plays a pivotal role in CTL-mediated tumor cytotoxicity by enhancing immune recognition and destruction of tumor cells. It upregulates MHC class I molecules on tumor cells, improving antigen presentation and making tumor cells more visible to CD8⁺ T cells [47, 48]. Furthermore, IFNγ sensitizes tumor cells to apoptosis by increasing the expression of proapoptotic caspases. It also enhances the cytolytic activity of CD8⁺ T cells by promoting the expression of perforin, granzymes, and Fas ligands, which are critical for inducing tumor cell apoptosis [49, 50].

Consistent with these functions, Lau et al. [51] demonstrated a critical role for IFNγ in tumor control in vivo, showing that the loss of IFNγ in the host immune compartment severely compromises tumor control, whereas IFNγ receptor–deficient tumor cells can still be efficiently eliminated in an IFNγ-competent immune environment. These findings underscore the importance of immune cell–derived IFNγ in antitumour immunity. Building on this framework, we propose that IFNγ released locally at the immunological synapse acts as a rapid sensitizing signal that primes tumor cells for CTL-mediated killing rather than functioning as an independent cytotoxic effector. Consistent with this model, we observed that P815 cells are relatively insensitive to global IFNγ stimulation, suggesting that their cytotoxic responses may preferentially reflect localized or rapid IFNγ-mediated effects at synapses. In contrast, other tumor cell lines, such as A20 and B-cell–derived cancers, exhibit increased Fas expression and increased susceptibility to IFNγ-dependent killing. Neutralization experiments further confirmed that synaptic IFNγ amplifies cytotoxic responses in synergy with granule-derived effectors rather than independently inducing tumor cell death. Moreover, our data indicate that IFNγ contributes to target apoptosis through an IFNγ–STAT1–dependent, caspase-3–mediated pathway, acting in parallel with the classical granule-mediated killing mechanism.

Effector T cells exhibit considerable plasticity [31, 52, 53]. While IFNγ expression is correlated with cytotoxicity, IFNγ-negative T cells maintain effector function via perforin, TNFα, and IL-2 [54]. The overexpression of IFNγ alters granule secretion patterns, favoring SCG release over MCGs, suggesting the dynamic adaptation of granule utilization in response to high cytokine production or acute inflammation [5]. Structural heterogeneity within CGs further supports this adaptability, with IFNγ distributed across SCGs and MCGs, which serve as reservoirs for soluble molecules, exosomes, and SMAPs [5]. Secreted particles from CD8⁺ T cells are highly heterogeneous and include double-positive particles containing both IFNγ and GzmB, as well as single-positive particles with either IFNγ or GzmB. The association of IFNγ-containing particles with various SMAP markers suggests a dynamic sorting mechanism reflecting immune plasticity.

Our findings reveal that IFNγ in activated effector T cells exists as two populations: a synapse-polarized “lytic” pool and a distal, nonlytic pool comprising both preexisting and newly synthesized IFNγ. IFNγ production and release occur continuously during synaptic engagement. The lytic pool is primarily stored in CGs and may be released at the synapse, whereas the nonlytic pool is generated independently in MVBs and released multidirectionally in a delayed manner, likely contributing to immunomodulatory signaling rather than direct cytotoxicity. This multidirectional secretion may facilitate distal immune modulation, which is consistent with IFNγ receptors being detected on extracellular vesicles [55]. This dual-pool organization explains why previous studies predominantly observed polarized secretion while overlooking multidirectional release, likely due to imaging limitations [27, 28].

Given that cytotoxic T cells are dedicated primarily to target cell killing, the release of cytotoxic granules represents a prioritized secretory event. In brefeldin A–treated cells, the release of newly synthesized IFNγ is inhibited, abolishing the distal IFNγ pool. Even as cytotoxic granules undergo replenishment for subsequent killing events [56], continuously produced IFNγ may be sorted into these refilled granules together with other lytic molecules.

Alternatively, some IFNγ may originate from other vesicular sources, such as the small vesicles detected by TIRFM and CLEM imaging. Several studies have documented the broad effects of IFNγ on antitumor immunity [28, 30, 41, 57], supporting the notion of a more widespread secretion pattern. Although the detailed sorting mechanism remains unresolved, our observations provide new insight into the canonical view that IFNγ is released exclusively at the immunological synapse [27].

Understanding IFNγ compartmentalization has translational relevance. In Munc13-4–deficient CTLs, the absence of synaptic release is compensated for by extrasynaptic secretion, demonstrating the adaptability of cytokine pathways. Engineered platforms such as “armored” CAR-T cells [58] could leverage this compartmentalization to fine-tune cytokine release and balance cytotoxicity and immunomodulation to increase tumor killing while minimizing systemic activation.

In conclusion, we identified lytic IFNγ stored within CGs, coreleased with GzmB at the immunological synapse, and identified a secondary nonlytic pool trafficked via MVBs for distal secretion. This dual-pool model highlights the functional flexibility of IFNγ in both cytotoxicity and immune modulation, providing a framework for engineering T cells with optimized effector functions and informing novel strategies in cancer immunotherapy.

## Materials and methods

### Mice

Wild-type (WT) mice were purchased from Charles River. Synaptobrevin2-mRFP knock-in (Syb2 KI) [35], granzyme B-mTFP knock-in (GzmB KI) [32], and Munc13-4 KO [37] mice were generated as described previously. All the transgenic and WT mice used in this study were on a C57BL/6N background, except for the GzmB-KI mice, which were on a C57BL/6J background. Mice of both sexes, aged 15–22 weeks, were used for the experiments. The animals were housed under standard conditions (22 °C, 50–60% humidity, and a 12-hour light/dark cycle). All the experimental procedures were conducted in compliance with the regulations of the state of Saarland (Landesamt für Verbraucherschutz, AZ.: 2.4.1.1 and 11/2021).

### Human CTLs

Human peripheral blood mononuclear cells (hPBMCs) were obtained from the blood bank of the University Hospital of the Saarland. The local ethics committee of the Saarland Medical Association (Ärztekammer des Saarlandes) approved the research with human material conducted for this study (Az. 98/15). Human CD8⁺ T cells were negatively isolated from the PBMCs of healthy donors via the Dynabeads FlowComp Human CD8 Kit. CD8⁺ T cells were activated and cultured under the same conditions as those described for mouse CTLs, with AIM-V medium supplemented with 10% FCS, 0.5% penicillin‒streptomycin, and 100 U/mL human recombinant IL-2 (Gibco) to support expansion. The cells were cultured at 37 °C in a 5% CO₂ atmosphere.

### Cell culture

Splenocytes were isolated from 15–22-week-old mice as described previously [5]. Naive CD8 + T cells were positively selected via the Dynabeads FlowComp Mouse CD8 Kit (Invitrogen) following the manufacturer’s protocol. Isolated CD8 + T cells were stimulated with anti-CD3/anti-CD28 activator beads (1:0.6 ratio) and cultured for 5 days in AIM-V medium (Invitrogen) supplemented with 10% FCS, 1% penicillin‒streptomycin (Invitrogen), and 50 µM 2-mercaptoethanol. The cells were cultured at a density of ~1 × 10⁶ cells/mL in 24-well plates. On day 2 poststimulation, 100 U/mL recombinant IL-2 (Gibco) was added to support T-cell proliferation. The resulting activated effector CTLs were used for subsequent experiments.

For human CTL cultures, CD8+ T cells were negatively isolated from the PBMCs of healthy donors via the Dynabeads FlowComp Human CD8 Kit (Invitrogen). CD8+ T cells were activated and cultured under the same conditions as those described for mouse CTLs, with AIM-V medium supplemented with 10% FCS, 1% penicillin‒streptomycin, and 100 U/mL human recombinant IL-2 (Gibco). P815 target cells were cultured in RPMI medium (Invitrogen) supplemented with 10% FCS and 1% penicillin‒streptomycin (Invitrogen). All the cell cultures were maintained at 37 °C with 5% CO₂.

### Mouse xenograft model of lung cancer

This model was constructed in compliance with the German legislation governing animal welfare and the principles outlined in the Guide for the Care and Use of Laboratory Animals (8th Edition, 2011). Protocols for these experiments were approved by the local animal protection committee (permit number: 23/2020). Twelve-week-old male C57BL/6 J mice (22–25 g; Charles River Laboratories) were anesthetized with isoflurane (5% for introduction, 2% for maintenance). A total of 1 × 10⁶ Lewis lung carcinoma LL/2 cells suspended in PBS were subcutaneously injected into the left flank of each animal. Twenty-eight days after tumor inoculation, the mice were euthanized, and the tumors were excised and fixed in 4% formalin for 24 hours for subsequent immunohistochemical analysis. Fixed tumor tissues were sectioned into 100 μm thick slices via a vibrating microtome (Leica VT1200 S). The sections were stained with anti-IFNγ-Alexa488 (BioLegend, clone XMG1.2), anti-CD8a-PE (BD; clone 53-6.7), and anti-GzmB-Alexa647 (BioLegend; clone GB11) antibodies.

### Plasmids

To generate serglycin expression constructs, the pMax-mSTX11-mNeonGreen [59] plasmid was used as the starting vector. The mouse serglycin coding sequence was amplified via PCR from cDNA derived from activated mouse CD8⁺ T cells and subcloned and inserted into the pMax-mSTX11-mNeonGreen backbone via EcoRI and BamHI restriction sites, thereby replacing the STX11 coding sequence and generating pMax-mSrgn-mNeonGreen. To generate pMax-mSrgn-mScarletI, the mNeonGreen coding sequence was replaced with mScarletI using the BamHI and XmaI restriction sites.

Mouse and human IFNγ expression constructs were generated via PCR amplification of IFNγ coding sequences from cDNA derived from activated mouse CD8⁺ T cells or human peripheral blood mononuclear cells (PBMCs), respectively. For mouse IFNγ constructs, the IFNγ coding sequence was amplified via primers containing KpnI and EcoRI restriction sites and subcloned and inserted into the pMax-GzmA-mCherry backbone, replacing the granzyme A coding sequence to generate pMax-mIFNγ-mCherry. To generate additional fluorescent fusion constructs, mouse IFNγ was subcloned and inserted into pMax-mSrgn-mScarletI or pMax-mSrgn-mNeonGreen backbones via EcoRI and BamHI restriction sites, replacing the serglycin coding sequence and yielding pMax-mIFNγ-mScarletI and pMax-mIFNγ-mNeonGreen, respectively. C-terminal IFNγ mutants were generated via site-directed mutagenesis via an NEB site-directed mutagenesis kit according to the manufacturer’s instructions. The basic C-terminal motif of mouse IFNγ (RKRKRSRC) was replaced with either a neutral sequence (AAAAASAC; IFNγ-A) or an acidic sequence (EEEEESEC; IFNγ-E). Mutagenesis was performed using pMax-mIFNγ-mScarletI or pMax-mIFNγ-mNeonGreen as template plasmids. For human IFNγ constructs, the IFNγ coding sequence was amplified from PBMC-derived cDNA via primers containing EcoRI and BamHI restriction sites and subcloned and inserted into the pMax-mSrgn-mNeonGreen backbone, replacing the serglycin coding sequence to generate pMax-hIFNγ-mNeonGreen. All plasmid constructs were verified by Sanger sequencing (Microsynth Seqlab, Göttingen, Germany).

### Flow cytometry

For flow cytometry analysis, 0.5 × 10⁶ day 4–5 effector CD8 + T cells were resuspended in D-PBS (Gibco) and incubated in the dark on ice for 30 minutes with the following cell surface markers: anti-CD44-PE (eBioscience, clone IM7, 1:200), anti-CD62L-APC (BD Pharmingen, clone MEL-14, 1:200), and anti-mIFNγ-Alexa488 (BioLegend, clone XMG1.2, 1:100). Day 5 human effector CD8 + T cells were treated with 5 µg/mL brefeldin A (Sigma; B6542) for 2 hours before fixation and staining with anti-hIFNγ-Alexa594 (BioLegend, clone B27, 1:200). Intracellular IFNγ staining was performed using a Transcription Factor Buffer Set (BD Pharmingen). Data acquisition was performed on a BD FACSAria III analyzer (BD Biosciences) via BD FACSDiva™ software. Flow cytometry data were analyzed with FlowJo v10.0.7 software.

### Killing assay

Day 3 effector WT or Munc13-4 KO CTLs (5 × 10⁴ cells) were cocultured with P815 target cells at the indicated T-cell–target ratios in 96-well U-bottom plates. P815 cells were prestained with 0.5 µM CellTrace Far Red (Invitrogen) for 10 minutes at room temperature (RT) and washed once with D-PBS before being cocultured with T cells. The cells were cultured in RPMI-based medium for 3 hours in the presence of an anti-mCD3 antibody (5 µg/mL) at 37 °C with 5% CO₂. To evaluate the contribution of IFNγ to killing, cells were treated with anti-IFNγ (Biolegend, clone XMG1.2) to neutralize secreted IFNγ in the medium or recombinant mouse IFNγ (Abcam) to increase soluble IFNγ levels. Following incubation, the cells were stained with 0.5 µg/mL propidium iodide (PI) and analyzed by flow cytometry. Dead target cells were identified as the APC⁺ PI⁺ population.

For STAT1 inhibition, P815 cells were preincubated with either 1 µM fludarabine phosphate [39] (Sigma, F9813) or 1 µM DMSO as a vehicle control for 12 h in RPMI-based culture medium. Pretreated cells were then loaded with 0.5 µM CellTrace Far Red and washed once with DPBS before the killing assay. A total of 5 × 10⁴ P815 cells per well were plated in a U-bottom 96-well plate with 2.5 µg of anti-CD3ε and 0.4 µM NucView Caspase-3 substrate (Biotium) to label apoptotic cells. WT CTLs were added to the wells at the indicated effector-to-target ratios for a 4 h killing assay at 37 °C and 5% CO₂. Apoptotic cells were identified as the APC⁺ CC3⁺ population.

To evaluate the synergistic effects of IFNγ with other lytic molecules, P815 cells were seeded at 1 × 10⁵ cells per well in 96-well V-bottom plates. The cells were preincubated with recombinant mouse IFNγ (10 ng/ml) overnight and subsequently treated with a sublytic dose (see the next methods section) of human perforin (hPerf) in combination with mouse granzyme B (mGzmB, 600 nM) for 4 h at 37 °C. After incubation, the cells were washed once with cold PBS and resuspended in binding buffer. Annexin V–FITC (Immunostep; ANXVF-200T) was added according to the manufacturer’s protocol, and the cells were incubated for 15 min at room temperature in the dark. PI (2 μg/ml) was added during the last 5 min of incubation. The samples were then washed twice with binding buffer and resuspended prior to acquisition. Flow cytometry was performed on a MACSQuant VYB cytometer (Miltenyi Biotec), and the data were analyzed via Kaluza software (Beckman Coulter). Cell death was quantified as (i) the percentage of Annexin V–positive cells and (ii) the increase in cell death (%) relative to basal levels in vehicle-treated controls. Control conditions (vehicle, perforin only, and granzyme B only) were included in each experiment.

### Determination of sublytic perforin dose

The sublytic dose of human perforin (hPerf) was determined by assessing PI uptake. Briefly, 5 × 10⁴ P815 cells were incubated with serial dilutions of hPerf for 15 min at 37 °C. After washing, the cells were stained with PI (2 μg/ml) and analyzed via flow cytometry. A concentration of hPerf that induced ~5–20% cell death was defined as sublytic and was used for subsequent assays.

### Total internal reflection fluorescence (TIRF) microscopy

For secretion analysis, day 5 effector mouse CTLs (mCTLs) isolated from GzmB KI mice and day 5 human CTLs were used. Mouse CTLs were transfected with mIFNγ-mCherry, while human CTLs were cotransfected with hIFNγ-mNeonGreen and hGzmB-mCherry via electroporation (Lonza kit). The cells were allowed to express transgenes for 12 hours at 32 °C in OptiMEM-based transfection medium supplemented with 10% FCS, 10 mM HEPES, 1% DMSO, and 1 mM sodium pyruvate [60] before TIRF microscopy. Two hours prior to imaging, the activator beads were removed from the cultures. For imaging, 3 × 10⁵ cells were resuspended in 30 µl of extracellular buffer (10 mM glucose, 5 mM HEPES, 155 mM NaCl, 4.5 mM KCl, and 2 mM MgCl₂) and allowed to settle for 1–2 minutes on anti-CD3-coated coverslips (30 µg/mL; clone 2c-11 for mouse, clone UCHT1 for human). The cells were then perfused with extracellular buffer containing calcium (10 mM glucose, 5 mM HEPES, 140 mM NaCl, 4.5 mM KCl, 2 mM MgCl₂, and 10 mM CaCl₂) to stimulate CG secretion. The TIRF microscopy setup (Visitron Systems GmbH) was based on an IX83 microscope (Olympus) equipped with a UAPON100XOTIRF NA 1.49 objective (Olympus), solid-state excitation lasers at 488 nm, 561 nm, and 647 nm, an iLAS2 illumination control system (Roper Scientific SAS), an Evolve-EM 515 camera (Photometrics), and a filter cube containing Semrock FF444/520/590/Di01 dichroic and FF01-465/537/623 emission filters. The system was controlled via Visiview software (version 4.0.0.11; Visitron Systems GmbH). Images were acquired at a frequency of 10 Hz with a 100 ms exposure time. Time-lapse series were analyzed via ImageJ or the FIJI package for ImageJ. CG secretion was quantified via ImageJ with the Time Series Analyzer plugin. A fusion event was defined as a fluorescence change within 300 ms accompanied by a diffusion cloud.

### Supported lipid bilayers (SLBs)

SLBs were prepared as previously described [61, 62]. Briefly, to prepare a clean glass chamber for SLB, glass coverslips prewashed with acid piranha and a plasma cleaner were mounted on sticky-Slide VI0.4 (Ibidi) to form 6 flow channels. Small unilamellar liposomes were prepared using 18:1 DGS-NTA(Ni) (790404C-AVL, Avanti Polar Lipids), 18:1 Biotinyl Cap (870282C-AVL, Avanti Polar Lipids), and 18:1 (D9-Cis) PC (850375C-AVL, Avanti Polar Lipids) in specific mixtures at a total lipid concentration of 4 mM. SLBs were allowed to form by incubating 50 µl of liposome suspension per flow channel for 20 min at 20 ± 2 °C. SLB were then washed with HEPES buffer containing 1 mM CaCl_2_ and 2 mM MgCl_2_ (HBS) and blocked with 1% human serum albumin (HBS/HSA) prior to functionalization. The SLBs were functionalized in two steps. First, 5 µg of streptavidin (S11226, Thermo Fisher Scientific) was added per channel for 10 min at 20 ± 2 °C, followed by three washes. Finally, 30 µg per channel biotinylated anti-mouse CD3ε (BD Pharmingen, clone 145-2C11) was linked to the streptavidin on the SLB, and 50 µg/mL 12-histidine-tagged mouse ICAM-1 was linked to nickel ions.

### Confocal live-cell imaging

Granule polarization to the synapse in CTLs targeting target cells was visualized via confocal microscopy (LSM 780, Zeiss) with a 63x Plan-Apochromat objective (NA 1.4; Zeiss). Briefly, day 5 IFNγ-mCherry-expressing GzmB-KI CTLs (1 × 10^5^) were cocultured with P815 target cells (2 × 10^4^) on polyornithine-coated glass coverslips to ensure less cell movement for imaging. The RPMI coculture medium contained 10 mM HEPES and 2.5 µg/mL anti-CD3ε antibody (BD Pharmingen, clone 145-2C11) to promote T-cell contact with target cells. Live imaging was performed at 37 °C. Images were acquired as z-stacks with 1 Hz acquisition over time. The total thickness of the stack was 6 µm, whereas the distance between individual slices was 1 µm. For TNFα and IL-2 localization, GzmB-tdTomato–derived CTLs were coincubated with P815 cells in the presence of 2.5 µg/mL anti-CD3ε antibody for 40 min. P815 cells were preloaded with CFSE (Invitrogen; 1:1000) to distinguish them from T cells before mixing. The cells were then fixed with 4% PFA and stained with anti–TNFα–Alexa Fluor 647 (BioLegend, clone MP6-XT22; 1:200) or anti–IL-2–APC (Invitrogen, clone JES6-5H4; 1:200) antibodies. To examine IFNγ localization within MVBs and cytotoxic granules (CGs), day 5 GzmB–tdTomato knock-in (KI) CTLs were harvested for organelle fractionation. Fractions 4 (enriched in MVBs) and 8 (enriched in SCGs) were fixed on coverslips and stained with anti-CD81 (Novus, clone 1D6; 1:200) and anti-IFNγ (BioLegend, clone XMG1.2; 1:100) antibodies.

### Structured illumination microscopy (SIM)

For colocalization studies, T cells were fixed with ice-cold 4% paraformaldehyde (PFA) in D-PBS (Thermo Fisher Scientific) for 20 minutes, permeabilized with 0.1% Triton X-100 in D-PBS for 20 minutes, and blocked for 30 minutes in the same solution containing 2% bovine serum albumin (BSA). The cells were stained with anti-hIFNγ-Alexa594 (BioLegend, clone B27, 1:200), anti-mIFNγ-Alexa647 (BioLegend, clone XMG1.2, 1:100), and anti-GzmB-Alexa647 (BioLegend, clone GB11, 1:100) antibodies. For the detection of secreted SMAP on SLBs, 0.5 × 10⁶ T cells were seeded per ibidi channel and incubated for 90 minutes at 37 °C with 5% CO₂ to allow synapse formation and SMAP secretion. The cells were washed five times with cold D-PBS to remove unbound cells while retaining deposited SMAPs. Deposited SMAPs were fixed with 2% prewarmed PFA for 2 minutes at 37 °C, washed three times with D-PBS, and stained with wheat germ agglutinin (WGA)-Alexa647 or WGA-Alexa488 (1 µg/mL) for 30 minutes at RT. SMAPs were then permeabilized with 0.05% Triton X-100 for 5 minutes, blocked with 2% BSA for 30 minutes, and stained with anti-GzmB-Alexa647. For analysis of IFNγ intracellular distribution and distal membrane secretion, WT and Munc13-4 KO T cells transfected with IFNγ-mCherry or human CTLs transfected with IFNγ-mNeongreen were seeded on anti-CD3-coated glass coverslips for 30 or 60 minutes at 37 °C with 5% CO₂. The cells were fixed with 4% PFA for 5 minutes and stained with WGA-Alexa488 (1 µg/mL) for 30 minutes at room temperature to mark the plasma membrane (PM). The IFNγ deposited on the coverslip was imaged to confirm distal membrane secretion. For quantification of the density of mouse IFNγ particles, cell-free areas were manually selected on the basis of brightfield images via ImageJ. Particles within the field of view containing transfected cells were counted and normalized to the selected area (µm²). For human IFN-γ particle analysis, owing to the low transfection efficiency, the transfected cells were not in close proximity to each other in the images. Therefore, particles were manually counted within an approximately 30 µm radius around each transfected cell. The analysis results are reported as the number of particles per cell. For IFNγ intracellular distribution, the cells were washed twice with D-PBS, permeabilized, blocked, and stained with anti-GzmB-Alexa647 (BioLegend, clone GB11, 1:100). For the inhibition of protein transport, day 4 mouse CTLs were transfected with the IFNγ–mCherry plasmid. After 6 h, the cells were treated with 1 μM brefeldin A (Sigma‒Aldrich, B6542) and incubated at 37 °C for 2 h. The cells were then washed once with RPMI-based culture medium and plated on anti-CD3–coated coverslips to induce synapse formation and vesicle secretion. The cells were fixed after 30, 60, and 120 min for imaging.

For T-cell-targeted conjugates, 0.5 × 10⁴ T cells were mixed with calcein blue AM (Invitrogen)-labeled P815 target cells (1 × 10⁴) on polyornithine-coated coverslips in RPMI-based medium supplemented with 5 µg/mL anti-mCD3 (BD Pharmingen, clone 145-2C11). The conjugates were fixed after 30 or 60 minutes with 4% PFA and stained with WGA-Alexa488 to mark the PM. For human CTLs, day 5 effector T cells were cocultured with P815 target cells in the presence of 5 µg/mL anti-hCD3 (Biolegend, clone UCHT1), fixed after 1 or 2 hours, and stained with WGA-Alexa488. Following permeabilization and blocking, the cells were stained with anti-hIFNγ-Alexa594 (BioLegend, clone B27, 1:200) and anti-GzmB-Alexa647 (BioLegend, clone GB11, 1:100) antibodies. The samples were mounted in Mowiol-based mounting medium. All the experiments were performed on a Zeiss Elyra PS.1 microscopic SIM system with Zen 2010 software for device control and high-resolution image processing. Images were acquired as z-stacks with 0.2-µm intervals through the entire cell. Single-plane and maximum intensity projections were used to illustrate vesicle colocalization.

For IFNγ sorting experiments, day 4 activated WT CTLs were cotransfected with IFNγ–mScarlet or the corresponding C-terminal mutants together with mSerglycin–mNeonGreen to assess the colocalization of IFNγ and serglycin by SIM. To control for potential overexpression artifacts, an additional set of experiments was performed using day 4 activated granzyme B–tdTomato knock-in CTLs transfected with IFNγ–mNeonGreen or the corresponding mutants to analyze the colocalization of IFNγ with granzyme B. For transfection, 6 × 10^6^ CTLs were electroporated with 2 μg IFNγ–mScarlet and 2 μg mSerglycin–mNeonGreen plasmids or with 2 μg IFNγ–mNeonGreen alone. The cells were incubated for 8 h at 37°C to allow protein expression, after which they were fixed and imaged directly via SIM.

For CLEM, images of cells embedded in 100 nm ultrathin Lowicryl sections were acquired with excitation wavelengths of 405, 488 and 561 nm. Almost the entire field of view of a 200-mesh grid (approximately 90 µm^2^) could be observed with a 63× Plan-Apochromat objective by SIM, allowing a perfect orientation relative to the grid bars. After adjusting the highest and lowest focus planes for z-stack analysis in brightfield, images were recorded with a step size of 100 nm. The fluorescence images were excited at 405 nm to visualize the DAPI signal. A wavelength of 488 nm was used for visualization of GzmB, and a wavelength of 561 nm was used for IFNγ. The DAPI image (405 nm wavelength) was used to identify the nuclei, the plasma membranes of the CTLs, and the image plane. Three to ten images were recorded with a step size of 100 nm to scan the cells of interest. All the images were acquired with a 63× Plan-Apochromat (NA 1.4). For fluorescence analysis of isolated cytotoxic organelles from Syb2-KI CTLs, they were centrifuged on gelatin-coated coverslips from fractions 6 (MCGs) and 8 (SCGs) and permeabilized with 0.005% Triton-X-100 in Dulbecco’s PBS following blocking in PBS containing 2% BSA. Organelles were stained with anti-GzmB-Alexa647 (BioLegend, clone GB11, 1:100) and primary anti-mouse IFNγ antibodies (BioLegend, clone XMG1.2, 1:100), followed by incubation with an anti-rat IgG1-Alexa488 secondary antibody (Invitrogen). IFNγ, Syb2-mRFP, and GzmB were visualized at 488 nm, 561 nm, and 642 nm, respectively.

### Cell homogenization and subcellular fractionation

Cytotoxic granule isolation was performed as previously described [36] and adapted for the isolation of MVBs. A total of 0.8–1.2 × 10^8^ activated CD8^+^ T cells from one Syb2 KI mouse were harvested and washed once in buffer (Invitrogen, PBS with 0.1% BSA and 2 mM EDTA) before resuspension in 2 mL of homogenization buffer (300 mM sucrose, 10 mM HEPES (pH 7.3), and 5 mM EDTA (pH 8.0)) supplemented with protease inhibitors (3 mM Pefabloc, 10 µM E64 and 10 µg of pepstatin A). The cell suspension was transferred into a prechilled cell disruption bomb (Parr 1019HC T304 SS) connected to a nitrogen source. After 25 minutes of equilibration at 55 bari, the nitrogen pressure was released, and the cell homogenate was collected. The cell lysate was centrifuged for 10 minutes at 1000 × g at 4 °C to pellet unbroken cells and partially disrupted cells and nuclei. The resulting postnuclear supernatant was layered on top of a discontinuous sucrose density gradient with 0.8, 1.0, 1.2, 1.4 and 1.6 M sucrose in 10 mM HEPES with 5 mM EDTA with protease inhibitors as described previously (pH 7.3), with 2 mL for each fraction. For additional MVB isolation, 2 ml of the 0.8 M sucrose fraction was replaced with 1 ml of 0.5 M sucrose and 1 ml of 0.8 M sucrose (corresponding to fractions 3 and 4). After ultracentrifugation at 100,000 × *g* for 90 minutes at 4 °C in a SW40Ti rotor (Beckmann), twelve 1 mL fractions were collected from the top of the gradient and supplemented with fresh protease inhibitors. For fluorescence analysis, 1 mL each of fractions 6 and 8 was diluted in 10 mL D-PBS and centrifuged for 30 minutes at 10,000 × *g* at 4 °C in an SW41Ti rotor (Beckmann) on gelatin-coated coverslips.

### RNA isolation, cDNA synthesis, and RT–qPCR

WT or Munc13-4 KO CD8⁺ T cells (1 × 10^6^ cells per well) were stimulated on anti-CD3-coated plates (30 µg/mL) or cultured on polyornithine (Sigma)-coated plates as an unstimulated control in a 24-well plate for 10 or 20 hours. Total RNA was extracted via TRIzol reagent (Thermo Fisher Scientific) following the manufacturer’s protocol. Briefly, 1 × 10⁶ CD8⁺ T cells were homogenized in 500 µl of TRIzol reagent, followed by the addition of 100 µl of chloroform. The samples were subsequently centrifuged at 12,000 × *g* for 15 minutes at 4 °C, after which the mixture was separated into three distinct phases, with the RNA remaining exclusively in the upper aqueous phase. The aqueous phase was carefully transferred to a fresh tube without disturbing the interphase, and the RNA was precipitated by mixing with an equal volume of 100% isopropanol. The RNA pellet was washed once with 75% ethanol, air-dried, and dissolved in DEPC-treated H₂O. The quality and quantity of the extracted RNA were assessed via a NanoDrop spectrophotometer. The isolated RNA was subsequently reverse transcribed into complementary DNA (cDNA) via the ABScript II cDNA First-Strand Synthesis Kit (ABclonal) in accordance with the manufacturer’s instructions. Briefly, 1 µg of RNA was mixed with a reaction mixture containing random primer mixture, 10 mM dNTPs and the ABScript II enzyme. The mixture was first incubated at 25 °C for 5 minutes, followed by incubation at 42 °C for 1 hour. Enzyme inactivation was achieved by heating the mixture to 80 °C for 5 minutes. RT‒qPCR was performed via the use of Genious 2X SYBR Green Fast qPCR Mix (ABclonal) on a Bio-Rad CFX96 Touch Real-Time PCR Detection System. A total of 10 ng of cDNA was used as a template in each reaction. Amplification was performed according to the manufacturer’s protocol via specific primers targeting Munc13-1 (forward: 5′-CAACTGGAATTACTTTGGC-3′, reverse: 5′-GCGAAGTCGTATAGTAGG-3′) and Munc13-4 (forward: 5′-GCCATTCTGCCCCTGATGAAAT-3′, reverse: 5′-ACCTCCACCAGCACAGTAAG-3′). TBP1 (Qiagen, QT00198443) was used as an internal reference gene to normalize target gene expression. Relative gene expression levels were quantified via the 2^(-ΔCt) method.

### ELISA

WT or Munc13-4 KO CD8⁺ T cells (1 × 10^6^ cells per well) were stimulated on anti-CD3-coated plates (BD Pharmingen, clone 145-2C11, 30 µg/mL) in 24-well plates containing 1 mL of AIMV-based culture medium. Unstimulated control cells were plated on polyornithine-coated plates. The supernatants were collected at the indicated time points during 20 hours of incubation at 37 °C. IFNγ concentrations were quantified via an IFNγ ELISA kit (Abcam, ab46081) according to the manufacturer’s instructions. Briefly, 100 µL of 1:10-diluted culture supernatant was added to IFNγ antibody-precoated 96-well plates and incubated for 1 hour to form antibody–antigen complexes. Following the washes, 100 µL of TMB substrate was added, and the mixture was incubated for 30 minutes at RT in the dark. The reaction was stopped with 50 µL of stop solution, and the absorbance was measured at 450 nm via a TECAN Infinite M200 Pro plate reader. Four independent coculture preparations were analyzed.

### Electron microscopy

Postembedding correlative light and electron microscopy (CLEM) of cryofixed mouse CTLs was performed as described previously [5]. Day 4 GzmB-mTFP KI CTLs were transfected with IFNγ-mCherry for 6–7 hours, and day 4 human effector CTLs were cotransfected with GzmB-mCherry and IFNγ-mNeonGreen for 8–10 hours at 32 °C before freezing. The cells were seeded on 1.4 mm sapphire discs coated with poly-L-ornithine (Sigma, 0.1 mg/mL) and anti-CD3 (BD Pharmingen, Clone145-2C11; 30 µg/mL) in flat specimen carriers (Leica) and vitrified in a high-pressure freezing system (EM PACT2, Leica). The frozen samples were cryo-transferred into the precooled (−130 °C) freeze-substitution chamber of the AFS2 (Leica). The temperature was increased from -130 to −90 °C for 2 h. Cryosubstitution was performed at −90 °C to −70 °C for 20 h in anhydrous acetone and at -70 to -60 °C for 24 h with 0.3% (wt/vol) uranyl acetate in anhydrous acetone. At -60 °C, the samples were infiltrated with increasing concentrations (30, 60 and 100%; 1 h each) of Lowicryl (3:1 K11M/HM20 mixture; Electron Microscopy Sciences). After 5 h of 100% Lowicryl infiltration, the samples were UV-polymerized at -60 °C for 24 h and for an additional 15 h while the temperature was increased linearly to 5 °C. The samples were stored in the dark at 4 °C until further processing. After the membrane carriers were removed, 100-nm ultrathin sections were cut via an EM UC7 (Leica) and collected on carbon-coated 200-mesh copper grids (Plano). One day after sectioning, the grid was stained with DAPI for 3 minutes (1/1000), washed and sealed between a coverslip and a glass slide for high-resolution SIM imaging. After fluorescence imaging, the grid was carefully removed, stained with uranyl acetate and lead citrate and recorded with a Tecnai12 Biotwin electron microscope. Only CTLs with well-conserved membranes, organelles and nuclei were analyzed and used for correlation. The DAPI signal of the nucleus was used to find the best overlap of the SIM image with the electron microscope image. The final alignment defines the position of the fluorescent signals within the cell of interest. The images were overlaid in CorelDRAW 2021.

### Statistical analysis

All the statistical analyses were performed via Igor Pro (v6.37) or SigmaPlot (v14.0). The data are presented as the means ± SEMs (or SDs where indicated). The statistical tests used for each experiment are specified in the corresponding figure legends. Unless otherwise stated, comparisons between two groups were performed via the Mann–Whitney U test, and multiple-group comparisons were analyzed via the Holm–Šidák post hoc test. All *p*-values were calculated via two-tailed tests with a 95% confidence interval. Statistical significance was denoted as *p < 0.05, **p < 0.01, and ***p < 0.001. All imaging data were analyzed with ImageJ or FIJI (version 15). Colocalization analyses were performed via the JaCoP plugin. Object-based colocalization was conducted with the DiAna plugin (ImageJ v52). Individual fluorescent spots were identified through an iterative segmentation procedure with a step value of 100, a size range of 15–700 pixels, and independently adjusted thresholds for each channel.

## Supplementary information

Supplementary information

Supplementary Video 1

Supplementary Video 2

Fig. 7E Raw Image 3D 30 Min

Fig. 7E Raw Image 3D 60 Min

Raw data

Figure S1

Figure S2

Figure S3

Figure S4

Figure S5

Figure S6

## Data Availability

The raw images and associated materials have been deposited in Zenodo 10.5281/zenodo.18414043). All other data supporting the findings of this study are available from the corresponding author upon reasonable request.
