# Supplementary material for: Lytic IFNγ is stored in cytotoxic granules and coreleased with granzyme B to mediate cytotoxic T lymphocyte killing
Source: Cell Mol Immunol. 2026 Mar 4;23(4):400–16. doi: 10.1038/s41423-026-01391-1 (PMC13035909; doi:10.1038/s41423-026-01391-1)
Supplement: Supplementary file 1 — Supplementary information [file 41423_2026_1391_MOESM1_ESM.pdf]

Supplementary information

Title: Lytic IFNγ is stored in cytotoxic granules and co-released with granzyme B to mediate cytotoxic T lymphocyte killing

Xuemei Li[1,2,#], Claudia Schirra[1,#], Marie-Louise Wirkner[1], Szu-Min Tu[1], Chin-Hsin Lin[1], Meltem Hohmann[1], Yuan Gu[3], Llipsy Santiago[4,5], Julian Pardo[4,5], Iñaki Arretxe[4], Nadia Alawar[1], Abed Alrahman Chouaib[1], Ute Becherer[1], Po-Hsien Lee[6], Hao-Jen Hsu[6], Matthias W. Laschke[3], Cosima T. Baldari[7], Michael L. Dustin[8], Jens Rettig[1], Elmar Krause[1]*, Hsin-Fang Chang[1]*

Summary:

The supplementary information includes two live-cell imaging videos of mouse and human CTLs secreting IFNγ and GzmB (corresponding to Fig. 2B and Fig. 5A); six supplementary figure detailing IFNγ expression, specificity of sorting to cytotoxic granules, secretion dynamics, transcriptional analysis, and vesicle polarization in WT and Munc13-4 KO CTLs; and two videos demonstrating the 3D images shown in Fig. 7E.

Supplementary Video 1, related to Figure 2: Live TIRF imaging of GzmB and IFN secretion by a mouse CTL. Day 5 mouse GzmB-mTFP KI effector CTLs (magenta) expressing IFNγ-mCherry (cyan) were plated on anti-CD3 antibody coated coverslips to induce synapse formation and granule release. Images were acquired at 5 Hz and compiled at 100 frames per second (fps).

Supplementary Video 2, related to Figure 5: Live TIRF imaging of GzmB and IFNγ secretion by a human CTL. Day 5 human effector CTLs expressing hIFNγ-mNeonGreen (cyan) and hGzmB-mCherry (magenta) were plated on anti-CD3 antibody coated coverslips to induce synapse formation and granule release. Images were acquired at 5 Hz and compiled at 100 frames per second (fps).

Supplemental Figure legends:

Figure S1. IFNγ sorting to cytotoxic granules is specific, related to Figure 1.

(A) C-terminal mutations in IFNγ reduce the predicted isoelectric point (pI) without altering the overall protein fold. Protein structures were predicted using AlphaFold3. Substituted amino acid residues at the C terminus are highlighted in wild-type IFNγ, the IFNγ-A mutant, and the IFNγ-E mutant. (B-C) Super-resolution SIM images and co-localization analysis of IFNγ with serglycin (Srg) in CTLs, quantified using Manders' and Pearson's correlation coefficients. Day 4 wild-type mouse CTLs were co-transfected with mScarletI-tagged IFNγ (WT, IFNγ-A, or IFNγ-E) together with a serglycin–mNeonGreen construct and fixed 8 h after transfection for SIM imaging. n = 23–33 cells per group from one mouse preparation. Scale bar: 5 μm. (D-E) SIM images and co-localization analysis of IFNγ with granzyme B (GzmB), quantified using Manders' and Pearson's correlation coefficients. Day 4 GzmB–tdTomato knock-in CTLs were transfected with mNeonGreen-tagged IFNγ constructs (WT and mutants) and fixed 8 h after transfection for SIM imaging. Super-resolution reconstructed images were used for quantitative analysis. n = 12–21 cells per group from two independent mouse preparations. Scale bar: 5 μm. Data are presented as mean ± s.e.m. Statistical significance was assessed using the Holm–Šidák post hoc test: $*P < 0.05$, $**P < 0.01$, $***P < 0.001$. (F) Confocal images of CTLs derived from the GzmB-tdTomato KI reporter mouse in contact with P815 target cells. P815 cells were preloaded with CFSE to distinguish them from T cells. T cells and target cells were co-incubated for 40 min before fixation. Fixed cells were stained with anti–TNF-α or anti–IL-2 antibodies to assess colocalization with $GzmB^+$ cytotoxic granules. Images were acquired at 0.5 μm intervals, and maximum projection images are shown. Scale bar: 10 μm.

Figure S2. Endogenous mouse IFNγ expression and localization on SMAPs, related to Figure 3.

(A) Flow cytometry analysis of WT day 5 effector cells without restimulation (N=3). Histograms show unstained controls (gray line) and cells stained with anti-mIFNγ-Alexa647 antibody from three preparations (red lines). (B) Quantification of IFNγ$^+$ cell populations from (A). (C) SIM images of isolated SMAPs from day 5 GzmB-tdTomato KI–derived CTLs. SMAPs were isolated from Fraction 6, which is enriched for MCGs in the cellular organelle fractionation. The isolated SMAPs containing GzmB (red) were fixed and stained with anti–IFNγ antibody (green) under permeabilized and non-permeabilized conditions. Scale bar: 1 μm.

Figure S3. Day 5 human effector CTLs retain IFNγ expression without additional restimulation, related to Figure 4.

(A) Flow cytometry analysis of day 5 effector CTLs from five donors. Cells were treated with Brefeldin A for 2 h, fixed, and stained with anti-hIFNγ-Alexa594 antibody. Histograms show unstained controls (gray line) and anti-IFNγ antibody-stained cells from 5 individual donors (red lines). (B) Quantification of IFNγ$^+$ cell populations from (A).

Figure S4. IFNγ mRNA transcription, secretion, and functional analysis in WT and Munc13-4 KO CTLs, related to Figure 6.

(A) Quantitative RT-PCR (qRT-PCR) was performed to analyze IFNγ transcription in WT and Munc13-4 KO day 5 effector CTLs (N=3). (B) IFNγ gene transcription in day 5 effector WT and Munc13-4 KO cells with or without CD3 restimulation. Cells were plated on polyornithine-coated (unstimulated control) or anti-CD3-antibody coated plates for the indicated times within 10 hours. mRNA was extracted from these cells for qRT-PCR analysis (N=3). (C) Constitutive release of IFNγ in WT and Munc13-4 KO effector CTLs. Day 5 effector cells were plated on polyornithine-coated plates. Supernatants from these cell groups were collected at the indicated time points within 20 hours. An ELISA assay was performed to measure the amount

of released IFNγ (N=3). (D) P815 target cells were treated with mouse recombinant IFNγ to evaluate its cytotoxicity. Dead cells were stained with PI and analyzed by FACS (N=3).

Figure S5. Overexpression of IFNγ in effector CTLs leads to increased constitutive secretion in the absence of CD3 stimulation, related to Figure 6. Day 5 effector WT cells were transfected with IFNγ-mCherry and incubated for 15 hours before the first supernatant collection (0 h). After 24 hours, the supernatant was collected again from the same culture. An ELISA assay was performed to measure the amount of released IFNγ. Data were collected from one experiment with three technical replicates.

Figure S6. Polarization of IFNγ$^+$ granules to the distal membrane in sustained synapses of mouse CTLs, related to Figure 7. (A-B) SIM images of day 5 effector WT and Munc13-4 KO CTLs fixed after 30 minutes (B) and 60 minutes (B) on anti-CD3-antibody coated coverslips. CTLs were transfected with IFNγ-mCherry (cyan) and stained with WGA (yellow) to label the plasma membrane. 3D reconstructed images show lateral views of granule polarization at the synapse, with (upper panels) and without (middle panels) plasma membrane labeling. White arrows highlight IFNγ$^+$ vesicles located at the synaptic layer (position 1) and distal membrane layer (position 2). Corresponding 2D xy images show single planes of the synaptic layer (position 1, lower left panel) and the distal membrane region (position 2, lower right panel). Small white arrows in the distal membrane region indicate IFNγ$^+$ vesicles attached to the plasma membrane at 60 minutes. Scale bar, 5 µm. (C) SIM images of day 5 mouse CTLs expressing IFNγ–mCherry, plated on anti-CD3–coated coverslips and incubated for the indicated times. Cells were fixed and stained with WGA–647 to label the plasma membrane. Maximum intensity projection (MIP) images are shown for representative cells, along with single image sections of the synaptic and distal planes. Cells were pretreated with Brefeldin A to inhibit protein transport, with DMSO-treated cells serving as controls. White arrows indicate small individual IFNγ$^+$ vesicles attached to the plasma membrane, and orange arrows indicate IFNγ$^+$ cytoplasmic aggregates resulting from Brefeldin A treatment. Scale bar: 5 µm. (D) SIM images of isolated MVBs and SCGs from day 5–activated GzmB-tdTomato CTLs. Cellular

organelle fractionation yielded an MVB-enriched fraction (fraction 4) and a classic single-core cytotoxic granule (SCG)-enriched fraction (fraction 8). These fractions were fixed and stained with anti-IFNγ. MVBs were additionally stained with anti-CD81 antibody to label exosomes. Scale bars: 2 μm.
