# Supplementary figures and images for: Lytic IFNγ is stored in cytotoxic granules and coreleased with granzyme B to mediate cytotoxic T lymphocyte killing
Source: Cell Mol Immunol. 2026 Mar 4;23(4):400–16. doi: 10.1038/s41423-026-01391-1 (PMC13035909; doi:10.1038/s41423-026-01391-1)

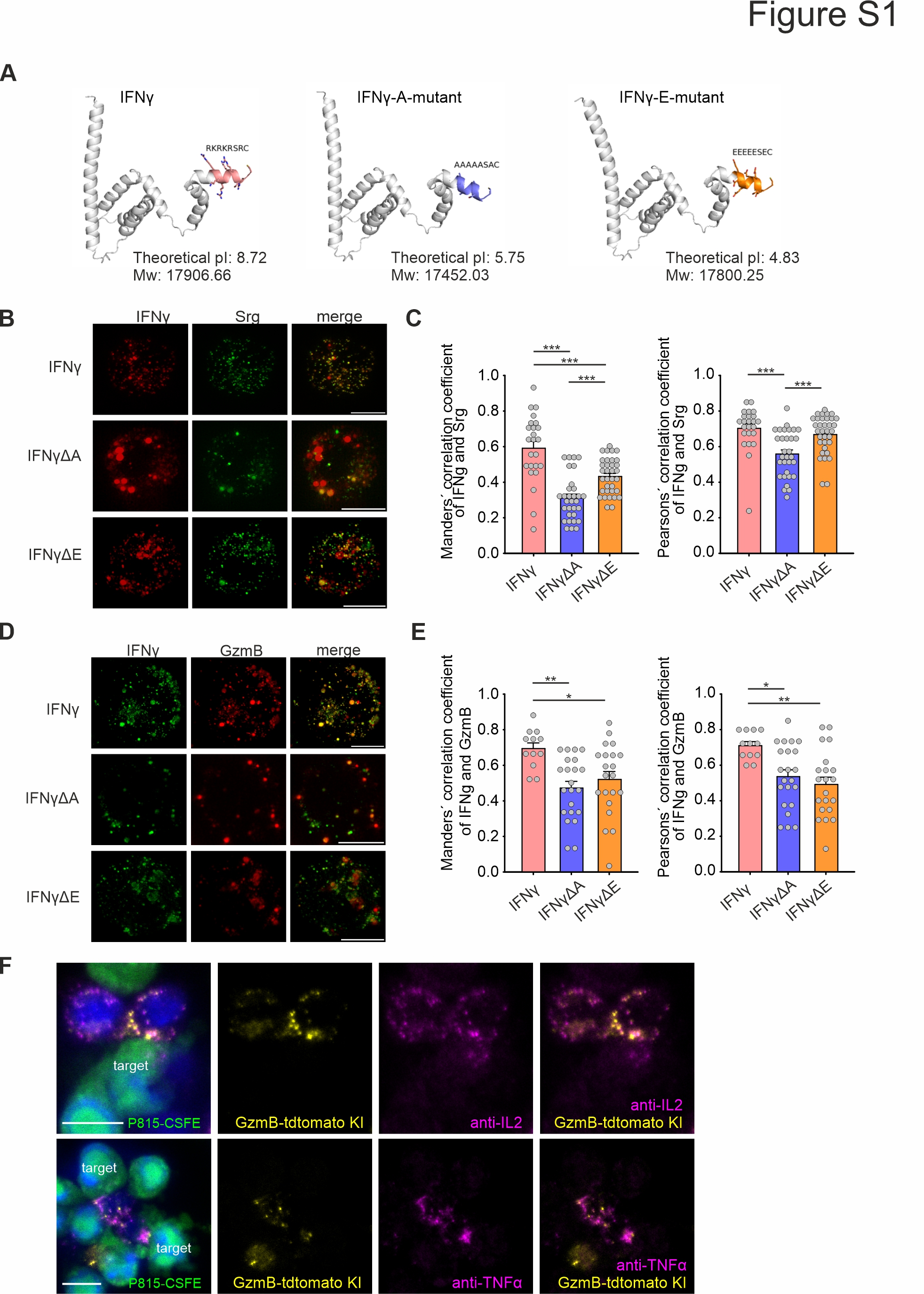

Supplement: Supplementary file 7 — Figure S1 [file 41423_2026_1391_MOESM7_ESM.jpg]

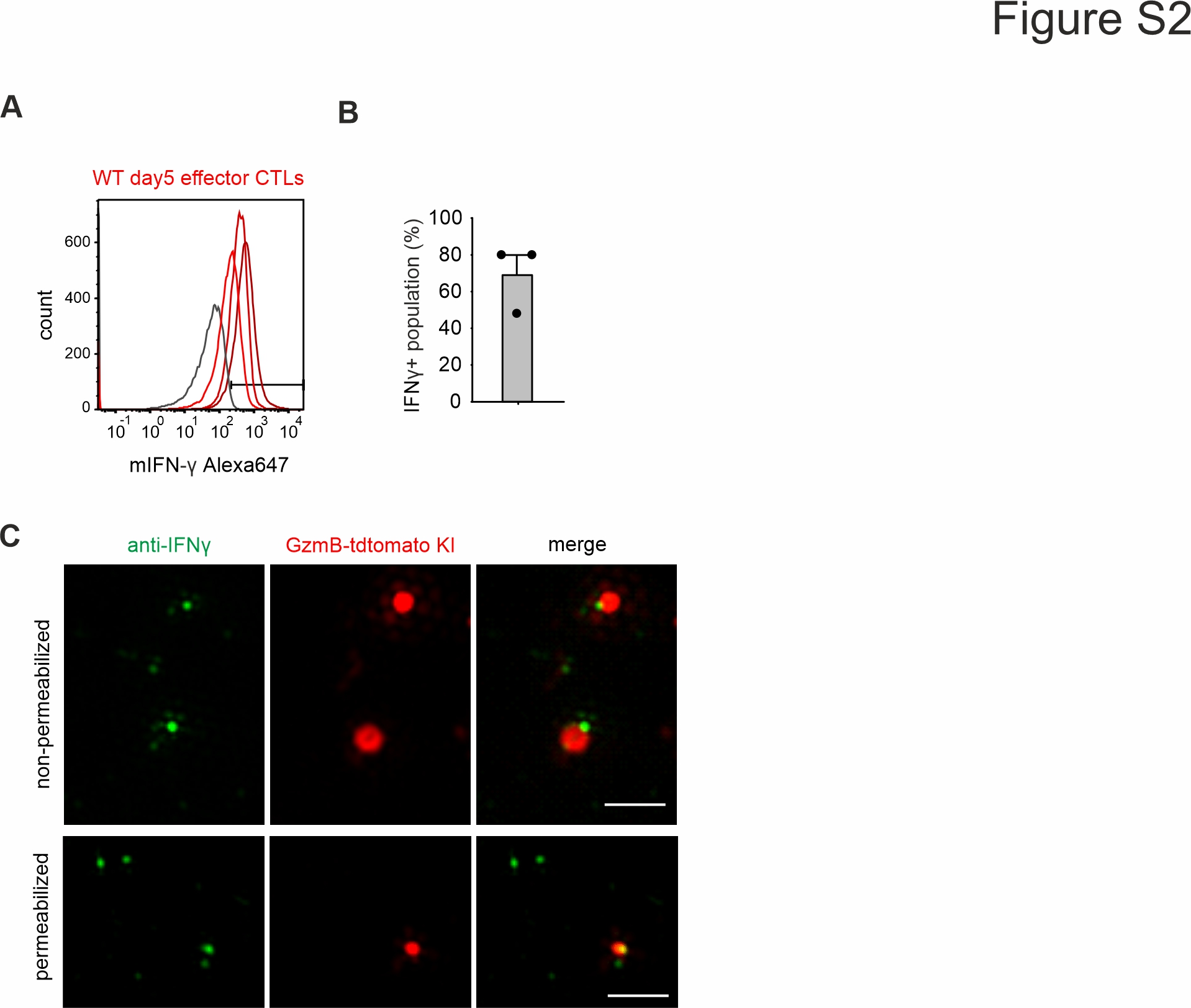

Supplement: Supplementary file 8 — Figure S2 [file 41423_2026_1391_MOESM8_ESM.jpg]

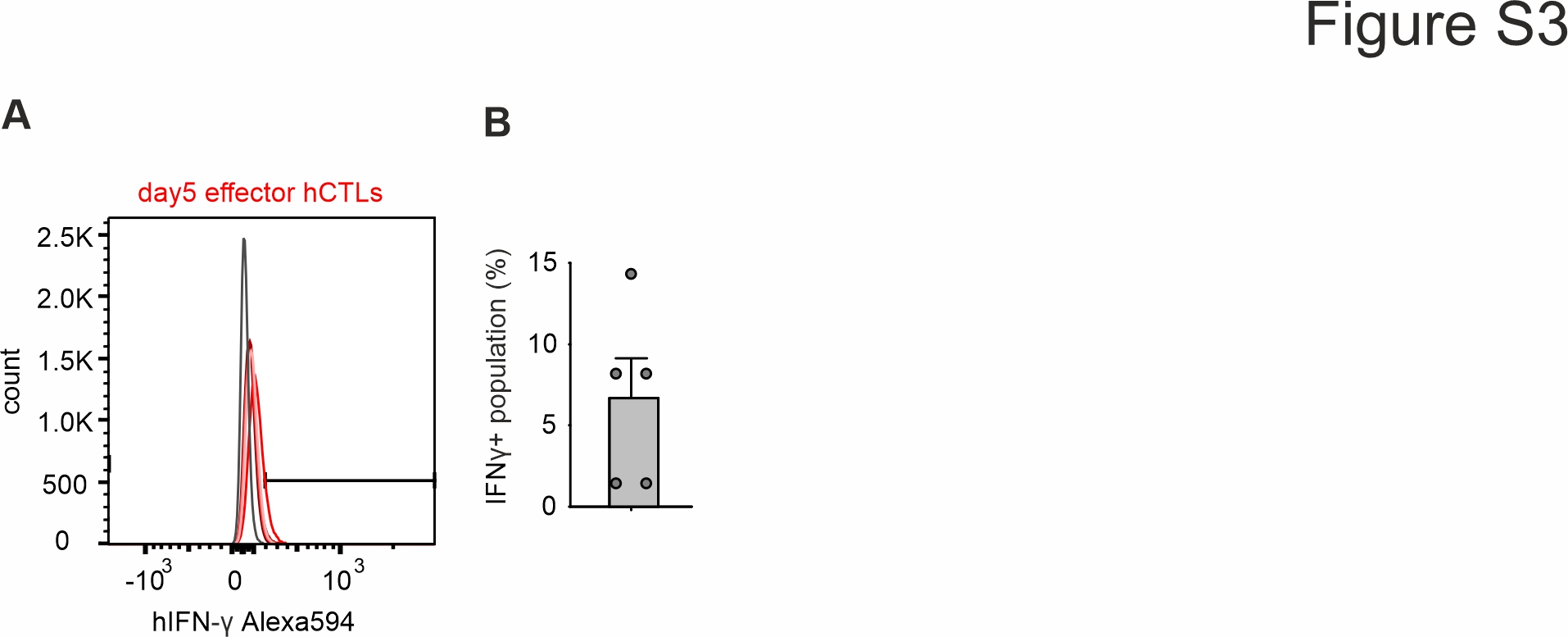

Supplement: Supplementary file 9 — Figure S3 [file 41423_2026_1391_MOESM9_ESM.jpg]

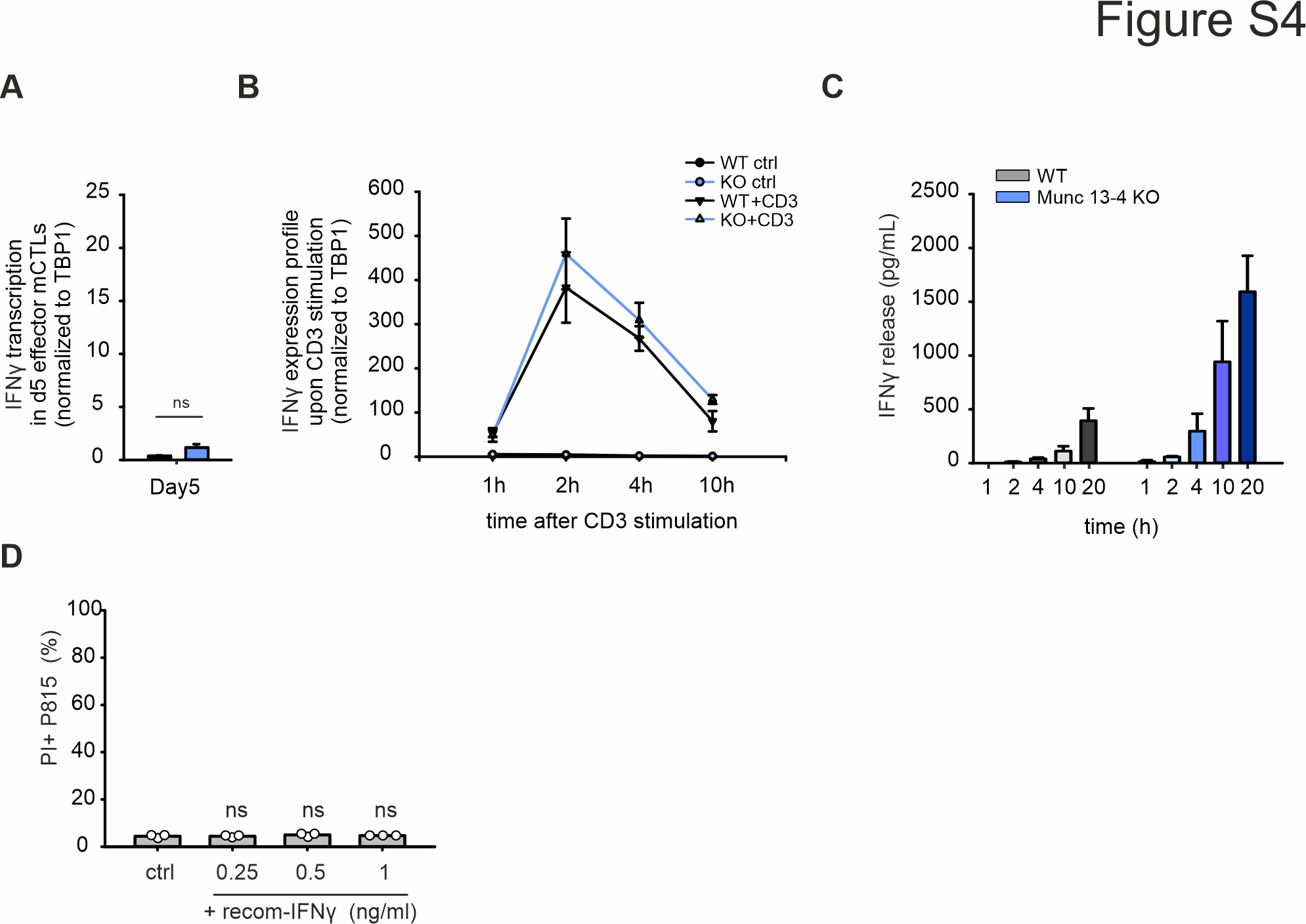

Supplement: Supplementary file 10 — Figure S4 [file 41423_2026_1391_MOESM10_ESM.jpg]

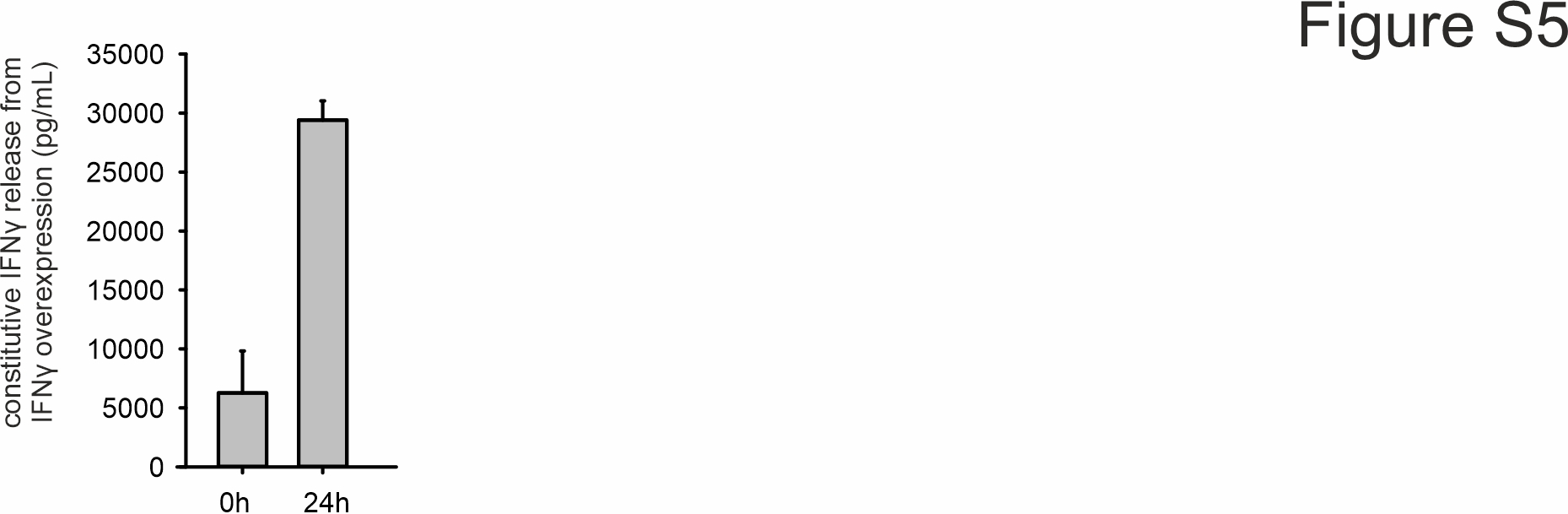

Supplement: Supplementary file 11 — Figure S5 [file 41423_2026_1391_MOESM11_ESM.jpg]

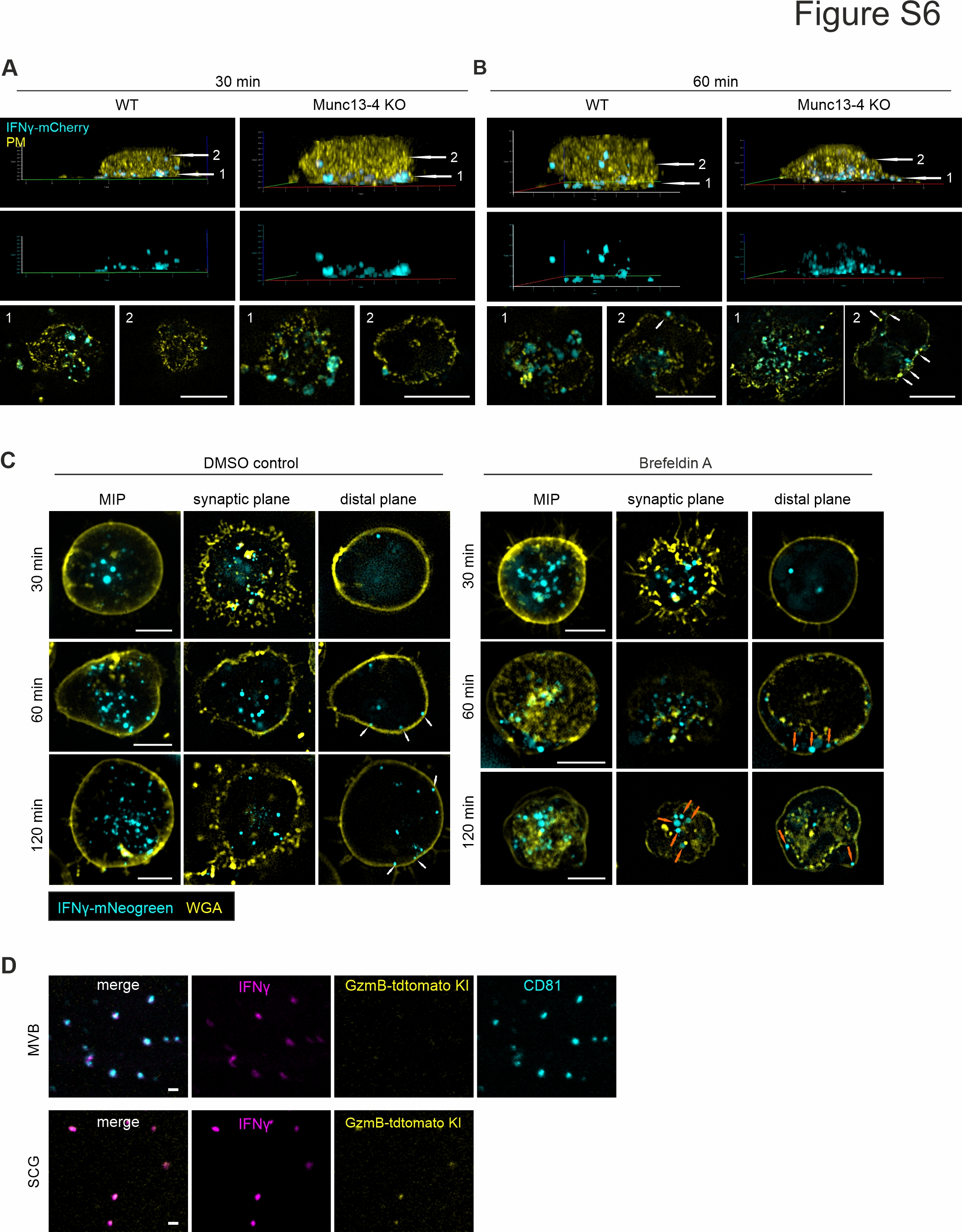

Supplement: Supplementary file 12 — Figure S6 [file 41423_2026_1391_MOESM12_ESM.jpg]
